# 1.3 V Inorganic Sequential Redox Chain with an All-Anionic Couple 1–/2– in a Single Framework

**DOI:** 10.1021/acs.inorgchem.1c01822

**Published:** 2021-10-24

**Authors:** Ana B. Buades, Clara Viñas, Xavier Fontrodona, Francesc Teixidor

**Affiliations:** † Institut de Ciència de Materials de Barcelona, Consejo Superior de Investigaciones Científicas, Campus Universitat Autonòma de Barcelona (UAB), 08193 Bellaterra, Spain; ‡ Departamento de Química and Serveis Tècnics de Recerca, Universitat de Girona, Campus de Montilivi, 17071 Girona, Spain

## Abstract

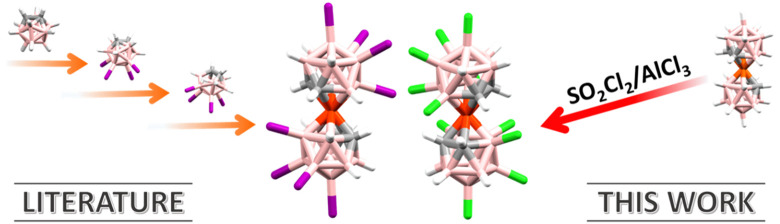

The relatively low symmetry of [3,3′-Co(1,2-C_2_B_9_H_11_)_2_]^−^ ([**1**]^−^), along with the high number of available substitution sites, 18 on the boron atoms and 4 on the carbon atoms, allows a fairly regioselective and stepwise chlorination of the platform and therefore a very controlled tuning of the electrochemical potential tuning. This is not so easily found in other systems, e.g., ferrocene. In this work, we show how a single platform with boron and carbon in the ligand, and only cobalt can produce a tuning of potentials in a stepwise manner in the 1.3 V range. The platform used is made of two icosahedra sharing one vertex. The *E*_1/2_ tuning has been achieved from [**1**]^−^ by sequential chlorination, which has given potentials whose values increase sequentially and linearly with the number of chloro groups in the platform. [**Cl**_**8**_**-1**]^−^, [**Cl**_**10**_**-1**]^−^, and [**Cl**_**12**_**-1**]^−^ have been obtained, which are added to the existing [**Cl-1**]^−^, [**Cl**_**2**_**-1**]^−^, [**Cl**_**4**_**-1**]^−^, and [**Cl**_**6**_**-1**]^−^ described earlier to give the 1.3 V range. It is envisaged to extend this range also sequentially by changing the metal from cobalt to iron. The last successful synthesis of the highest chlorinated derivatives of cobaltabis(dicarbollide) dates back to 1982, and since then, no more advances have occurred toward more substituted metallacarborane chlorinated compounds. [**Cl**_**8**_**-1**]^−^, [**Cl**_**10**_**-1**]^−^, and [**Cl**_**12**_**-1**]^−^ are made with an easy and fast method. The key point of the reaction is the use of the protonated form of [Co(C_2_B_9_H_11_)_2_]^−^, as a starting material, and the use of sulfuryl chloride, a less hazardous and easier to use chlorinating agent. In addition, we present a complete, spectroscopic, crystallographic, and electrochemical characterization, together with a study of the influence of the chlorination position in the electrochemical properties.

## Abstract 2

By sequential halogenation of [Co(C_2_B_9_H_11_)_2_]^−^ ([**1**]^−^) with chlorine, the [**Cl**_**8**_**-1**]^−^, [**Cl**_**10**_**-1**]^−^, and [**Cl**_**12**_**-1**]^−^ derivatives of [**1**]^−^ have been prepared and isolated. The *E*_1/2_ values increase sequentially and linearly with the number of chloro groups in the platform. If these potentials are added to the existing *E*_1/2_ values due to [**Cl-1**]^−^, [**Cl**_**2**_**-1**]^−^, [**Cl**_**4**_**-1**]^−^, and [**Cl**_**6**_**-1**]^−^ described earlier, a 1.3 V range is obtained. This allows tuning of the desired potentials for the purposes of nature.

## Introduction

Redox reactions are key for life both in nature,^1^ principally in respiration^2^ and photosynthesis,^3^ and in any device where electrons are the means to store, release, or generate energy.^4−11^

In most of the redox reactions in industry to produce bulk materials or compounds, no fine-tuning of the reduction or oxidation power is sought. However, this is not so when it is necessary to ensure the synergy with surrounding materials or compounds that can be affected by an excess of oxidizing or reducing power. *E*° tuning of man-made redox-reversible systems is largely based first on metals and second in ligands,^12−19^ Notice from this sentence that we emphasize metal-based redox-reversible systems. We will not deal with nonmetal-based systems because, for the case of boron clusters, these are derived from [CB_11_H_12_]^−^^20^ or [B_12_H_12_]^2–^.^21^ It is important to point out that nature succeeds in getting a wide range of potentials with few metals, few coordinating elements, and few ligands for the primary coordination spheres but requires the involvement of one or two extra spheres of influence to modulate *E*°.^15^ Some robust metal-containing scaffolds have been developed on which to tune the redox potential by the sequential addition of electron-donor or -acceptor groups or π acceptors. Some of the more studied scaffolds are due to ferrocene,^22,23^ or metal complexes, most commonly ruthenium, of polypyridyl ligands, e.g., bipyridine, 2,2′-bipyrimidine, 2,2′-bipyrazine, terpyridine, phenanthroline, and others.^24^ Their common factor is that they are usually outer-sphere electron-transfer octahedral complexes. A quite representative example of the type of *E*° tuning in these complexes is given by the ferrocene [FeC_10_H_10–*x*_Cl_*x*_] chloro derivatives for which brusque, the opposite of stepwise, numbers of chloro units exist, e.g., 10, 5, 2 and 1, which result in brusque *E*_1/2_ values, versus ferrocenium/ferrocene (Fc^+^/Fc) of 1.24, 0.77, 0.31, and 0.17 V, respectively. Still, nearly 1 V has been tuned on the same platform.^25,26^ All of these complexes are positively charged, e.g., [Fe(C_5_Cl_5_)_2_]^+^ or [Ru(bpy)_3_]^2+^. Indeed, despite the fact that ligands are either negative or neutral, very few chemically stable and robust anionic complexes are available ready for *E*° tuning. One could consider the couple [Fe(CN)_6_]^3–/4–^, or the polyoxometallates (POMs), e.g., Keggin [XW_12_O_40_]^*n*−^;^27^ however, these are difficult to tune, although efforts are being made for POMs.^28^

Thus, anionic metal-containing scaffolds that allow easy tuning with a wide span of voltages are not common. Also, what could be the advantage of using anionic scaffolds? In our opinion, if the reduced form of the redox couple is negative, it will have an increased tendency to release an electron, and if the oxidized form is negative, it will have less appetence for an electron. Plus, this can be easily spotted with the iodide/triiodide (I^–^/I_3_^–^) redox couple in dye-sensitized solar cells (DSSCs) in which both the oxidized and reduced partners are negative.^10,29,30^ Cobalt-^31^ and copper-based electrolytes,^32^ thiolate/disulfide,^33^ Fc/Fc^+^,^34^ hydroquinone/benzoquinone derivatives,^35^ and the redox couple TEMPO/TEMPO^+^^36^ all either have a partner whose charge is zero, have a partner with a positive charge, or have both partners with a positive charge. The success of a DSSC relies on the electrons preferring to move through the external circuit to meet the counter electrode rather than the electrons on the TiO_2_ surface recombining with the dye or oxidized electrolyte.^37^

We have already indicated that it is not simple to have metal-based robust redox couples based on a single scaffold that allow for a wide range of potentials. In this work, we show that this is becoming possible with the anionic cobaltabis(dicarbollide) [3,3′-Co(1,2-C_2_B_9_H_11_)_2_]^−^ scaffold (abbreviated as [**1**]^−^). This cluster displays interesting electrochemical and biological properties that have been thoroughly studied.^38−40^ Several [**1**]^−^ derivatives have been published with the aim of tailoring its properties and finding applications in many different fields of science. Some examples are neutron capture therapies,^41,42^ sensors,^43^ anticancer therapies,^44−48^ electron acceptors,^49^ and electroactive electrolytes among others.

The relatively low symmetry of [**1**]^−^, along with a high number of available substitution sites, allows a fairly regioselective and stepwise chlorination of the platform and therefore a very controlled tuning of the sought-after property, in this case potential tuning. Such characteristics are not easily found in other systems. On the other hand, a higher symmetry, as in many closo clusters, leads more easily to persubstitution but with more difficulty to a step-by-step process.^50^

**Figure 1:**
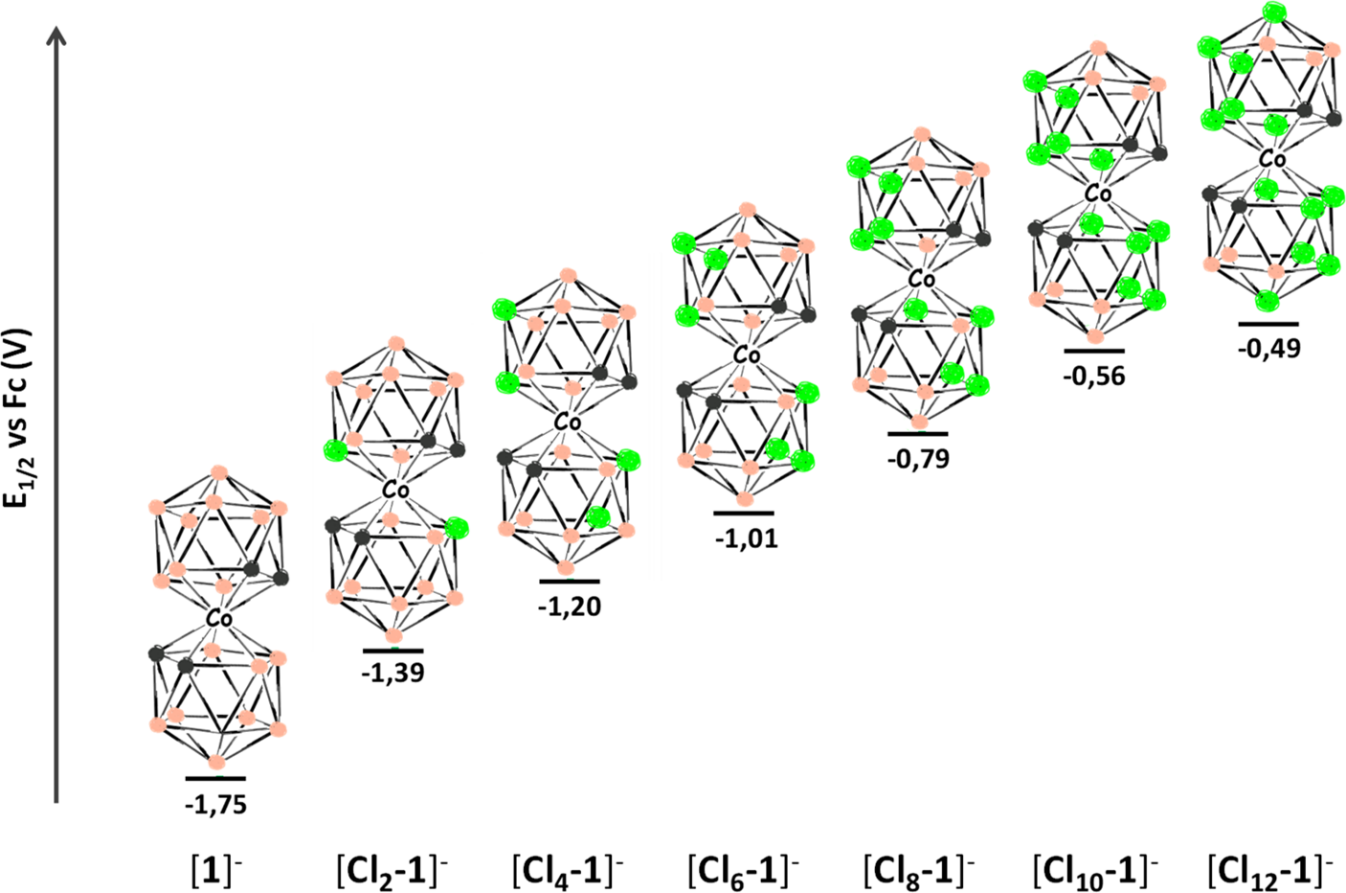
*E*_1/2_ scheme of the different chloro derivatives of cobaltabis(dicarbollide) in volts. B–H is represented by pink spheres, B–Cl by green spheres, and C–H by gray spheres.

We present here the three highest chlorinated species of [**1**]^−^, which will be named [**Cl**_**8**_**-1**]^−^, [**Cl**_**10**_**-1**]^−^, and [**Cl**_**12**_**-1**]^−^, corresponding to the number of chloro substituents on the scaffold, which span the voltages from −1.75 V for [**1**]^−^ to −0.49 V for [**Cl**_**12**_**-1**]^−^, versus Fc^+^/Fc in sequential chlorination steps, and very remarkably with very good electrochemical purity and high yield in simple one-pot reactions (Figure 1). This series is the widest range of sequentially tunable potentials on a single metal-containing anionic platform available today. Also, the range of potentials possible can be extended much further by keeping the same platform, changing the metal from cobalt to iron.

## Results and Discussion

### Synthesis

Since the synthesis of the first halogenated derivative of COSAN, the hexabromocobaltabis(dicarbollide),^51^ many strategies have been devised to develop halo derivatives of [**1**]^−^. The most advanced since that date is the development of iodo derivatives of [**1**]^−^, whose methodology requires the buildup of molecules from the components, so the synthesis of [1,5,6,10-I_4_-7,8-C_2_B_9_H_10_]^−^, followed by their complexation with CoCl_2_, yields [3,3′-Co(8,9,12,10-I_4_-1,2-C_2_B_9_H_7_)_2_)]^−^, which is the halo derivative of cobaltabis(dicarbollide) with the highest number of halo substituents produced until now.^52,53^

Chlorine gas was the most popular chlorinating agent for [**1**]^−^,^54−56^ with [3,3′-Co(8,9,12-Cl_3_-1,2-C_2_B_9_H_8_)_2_^53^ being the highest chlorinated [**1**]^−^ obtained as a pure compound since 1982. In 1980, sulfuryl chloride was used as a source of chlorine and solvent in the synthesis of [B_9_Cl_9_]^2–^.^57^ Sulfuryl chloride is less hazardous, cheaper, and easier to handle than chlorine gas and has been successfully applied as a chlorinating agent in organic chemistry.^58,59^ To achieve chlorination in boron clusters, solubilization of the cesium and tetramethylammonium (the most common) salts of the different boron clusters was needed, but these are not fully soluble in sulfuryl chloride. In 2010, a step forward was achieved by mixing acetonitrile with sulfuryl chloride to increase the solubility. This new method allowed the synthesis of pure [B_12_Cl_12_]^2–^,^60^ and afterward, the same methodology was used to obtain the hexachloroferrabis(dicarbollide)^61^ and tetrachloro-^62^ and hexachlorocobaltabis(dicarbollide).^63^ However, we did not succeed in going beyond with this mixture, even after several days of refluxing and repositioning of acetonitrile and sulfuryl chloride. Therefore, the combination of sulfuryl chloride with acetonitrile was not considered to be the best option. Instead, we have gone with H[**1**], which is more soluble in neat sulfuryl chloride.^64^ Because sulfuryl chloride has a relatively low boiling point, 69 °C, we aimed at increasing the reaction pressure to lower reaction times and increasing the reaction temperature. Stainless steel autoclaves, even lined with Teflon, were proven not to be suitable because extensive damage was caused by the generated chlorine gas at the autogenous pressure induced by external heating at 120 °C. We then moved to thick-walled glass pressure tubes with Ace-Thred poly(tetrafluoroethylene) bushing and FETFE O-ring. The O-rings were replaced every four experiments. These proved to be adequate for our purposes.

**Figure 2:**
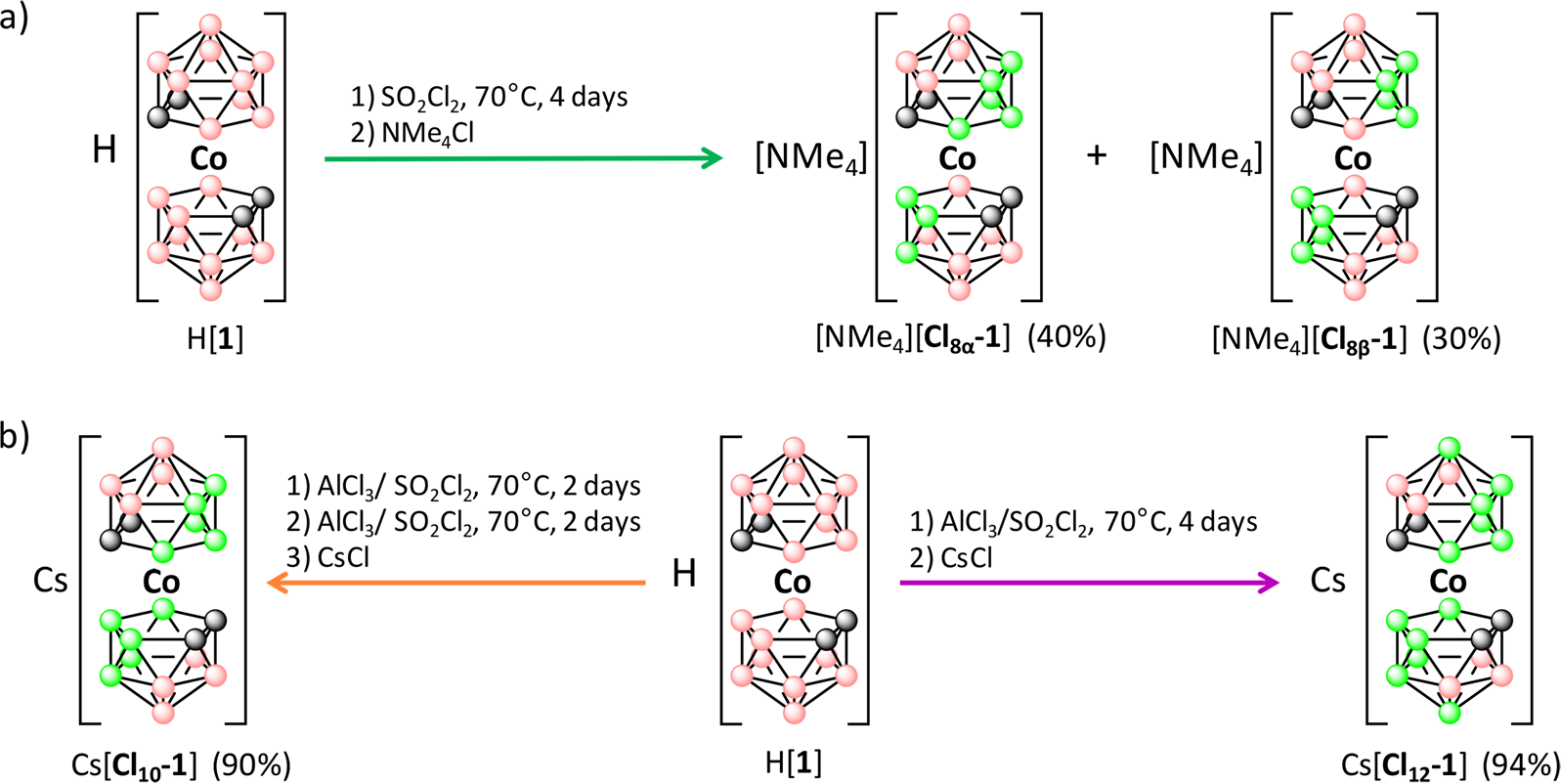
Reaction conditions for the synthesis of compounds (a) [**Cl**_**8α**_**-1**]^−^ and [**Cl**_**8β**_**-1**]^−^ and (b) [**Cl**_**10**_**-1**]^−^ and [**Cl_12_-1**]^−^. B–H is represented by pink spheres, B–Cl by green spheres, and C–H by gray spheres.

The reaction of H[**1**] with an excess of SO_2_Cl_2_ (650 equiv) in an Ace pressure tube at 70 °C for 4 days is a convenient route to synthesizing the octachloro derivative presented as an isomeric mixture of [3,3′-Co(4,7,8,9,12-Cl_5_-1,2-C_2_B_9_H_6_)(8′,9′,12′-Cl_3_-1′,2′-C_2_B_9_H_8_)]^−^ ([**Cl**_**8α**_**-1**]^−^) and [3,3′-Co(4,8,9,12-Cl_4_-1,2-C_2_B_9_H_7_)_2_]^−^ ([**Cl**_**8β**_**-1**]^−^) (Figure 2a). This turned out to be the maximum chlorination degree achievable by this method. Attempts to increase the chlorination degree by increasing the reaction time to a few weeks or using higher temperatures did not lead to notable amounts of [**Cl**_**9**_**-1**]^−^ or [**Cl**_**10**_**-1**]^−^) derivatives. It was then proven that a convenient and easy method leading to constitutionally, although not isomerically, pure [**Cl**_**8**_**-1**]^−^ was available. To increase the number of chloro groups in the molecule, a Lewis acid such as AlCl_3_ (1 equiv) was added to the reaction mixture, and this turned out to be the determining factor in obtaining a higher chlorination degree, leading to the production of [3,3′-Co(4,7,8,9,10,12-Cl_6_-1,2-C_2_B_9_H_5_)_2_]^−^ ([**C**_**12**_**-1**]^−^), the highest imaginable chlorinated redox-reversible couple (Figure 2b). In addition, the amount of SO_2_Cl_2_ was optimized to control the chlorination degree, leading to [3,3′-Co(4,7,8,9,12-Cl_5_-1,2-C_2_B_9_H_6_)_2_]^−^ ([**Cl**_**10**_**-1**]^−^). Thus, while the synthesis of [**Cl**_**12**_**-1**]^−^ requires a huge excess of SO_2_Cl_2_ (650 equiv), the synthesis of [**Cl**_**10**_**-1**]^−^ needs less chlorinating agent (65 equiv). The methodology consists of a mixture of 0.1 and 65 equiv of AlCl_3_ and SO_2_Cl_2_, respectively, with 1 equiv of H[**1**] in an Ace pressure tube at 70 °C for 2 days. Then the tube is open, and the solvent is removed under reduced pressure. Then, 0.1 and 65 equiv more of AlCl_3_ and SO_2_Cl_2_, respectively, are added to the solid reaction mixture, and the tube is closed again and is heated at 70 °C for 2 more days (Figure 2b).

### Characterization

All new compounds were characterized by ^1^H, ^1^H{^11^B}, ^13^C{^1^H}, ^11^B, ^11^B{^1^H} NMR, matrix-assisted laser desorption/ionization time-of-flight mass spectrometry (MALDI-TOF-MS) in the negative mode, elemental analysis, IR, and X-ray diffraction. The complete spectral information and crystallographic data can be found in the Supporting Information (SI). The IR spectra give us a qualitative analysis of the reaction by monitoring of the B–H band around 2600 cm^–1^. In addition, a comparison of the Fourier transform infrared (FTIR) spectrum of Na[**1**] with the spectra of [NMe_4_][**Cl**_**8**_**-1**], [NMe_4_][**Cl**_**10**_**-1**], and Cs[**Cl**_**12**_**-1**] unveils a band at 992 cm^–1^ corresponding to the B–Cl bond, and this band appears in other boron clusters in the literature such as [**B**_**12**_**Cl**_**12**_]^2–^, demonstrating the hypothesis.^65^ On the other hand, the MALDI-TOF-MS spectra in the negative mode provide faster and reliable information about the exact number of chloro substituents in the [**1**]^−^ skeleton. MALDI-TOF-MS of the 8, 10, and 12 chlorinated derivatives of [**1**]^−^ shows main peaks at *m*/*z* 598.7, 666.8, and 734.8 that correspond to [**Cl**_**8**_**-1**]^−^ in [NMe_4_][**Cl**_**8**_**-1**], [**Cl**_**10**_**-1**]^−^ in [NMe_4_][**Cl**_**10**_**-1**], and [**Cl**_**12**_**-1**]^−^ in Cs[**Cl**_**12**_**-1**] and represent 82, 90, and 97% of the sample, respectively. However, the MALDI-TOF-MS unveils a percentage of less than 10% of the side products corresponding to compounds with one chloro plus or less (see the SI).^56^

**Figure 3:**
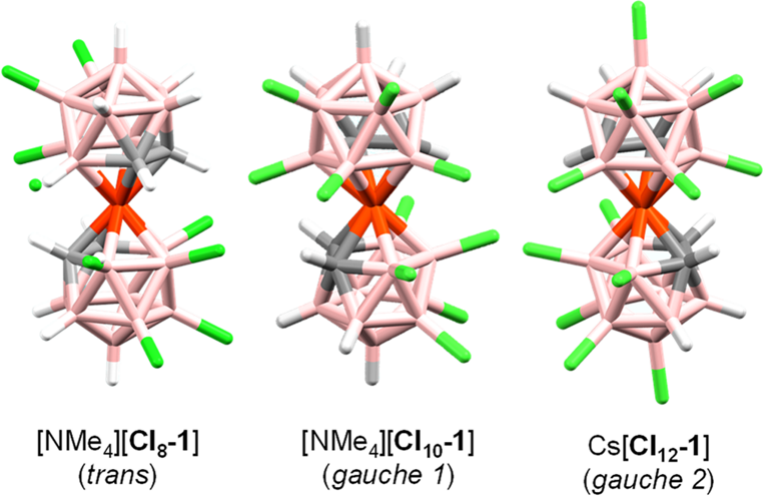
Crystal structures of [NMe_4_][**Cl**_**8**_**-1**], [NMe_4_][**Cl**_**10**_**-1**], and Cs[**Cl**_**12**_**-1**] (from left to right).

The study of the NMR spectra, together with X-ray diffraction, led us to unveil the exact positions of the chloro substituents. Suitable single crystals of [NMe_4_][**Cl**_**8**_**-1**] and [NMe_4_][**Cl**_**10**_**-1**] were obtained by slow evaporation in acetone; for Cs[**Cl**_**12**_**-1**], crystals were obtained in CH_2_Cl_2_, and as far as we are concerned, they are the highest halogenated derivatives of metallacarborane ever crystallized (Figure 3).

### Structures and Intermolecular Nonbonding Interactions

The representation of a chloro and a hydrogen atom in the B(4,4′) positions of [NMe_4_][**Cl**_**8**_**-1**] indicates the isomers present in the crystal (B is in pink, C in gray, H in white, Cl in green, and Co^3+^ in orange). X-ray analysis of [NMe_4_][**Cl**_**8**_**-1**] revealed the solid solution nature of the crystal due to the existence of two isomers in the same monocrystal. Specifically, the crystal demonstrated the existence of [NMe_4_][**Cl**_**8α**_**-1**] in 80% and [NMe_4_] [**Cl**_**8β**_**-1**] in 20% (Figure 3).

The structures of [NMe_4_][**Cl**_**8**_**-1**] and [NMe_4_][**Cl**_**10**_**-1**] show different types of intermolecular interactions because of its cation (see the SI). The dihydrogen bond C_Me_–H···H–B, which is the most abundant interaction in tetramethylammonium metallacarboranes,^66^ becomes less abundant in these derivatives because of the high chlorination degree of the molecules. Instead, the moderate hydrogen bonds C_Me_–H···Cl–B with distances from 2.745 to 2.911 Å dominate the intermolecular interaction for [NMe_4_][**Cl**_**10**_**-1**] with seven hydrogen bonds for each [NMe_4_]^+^. In contrast, the C_Me_–H···H–B interaction is more abundant than the C_Me_–H···Cl–B in the [NMe_4_][**Cl**_**8**_**-1**] structure because the chlorination degree is less. To that end, the most important interaction in the crystal structure of [NMe_4_][**Cl**_**10**_**-1**] is the double contact between Cl–B_B_(4) of the metallacarborane (B) and C_C_–H of the near metallacarborane (A), specifically, C_CA_(1)–H···Cl–B_A_(4) and B_CB_(4)–Cl···H–C_CA_(1′) with distances of 2.877 and 2.657 Å, respectively, with an angle of 51.4°.^67^ A study of the structure Cs[**Cl**_**12**_**-1**] reveals other types of interactions. The double contact B_A_(9′)–Cl···Cs···Cl–B_B_(9) with distances of 3.597 and 3.671 Å and the interaction of B_A_(7)–Cl···H–B_B_(11) and B_A_(8′)–Cl···H–B_C_(5) between three nearby metallacarboranes with distances of 3.118 and 2.999 Å, respectively, are the most significant. In addition, the structure shows interactions between two metallacarboranes and the CH_2_Cl_2_ solvent. Specifically, the solvent molecule interacts with the B(5)–H and B(9)–Cl positions of two metallacarboranes with distances of 2.251 and 2.902 Å, respectively. Finally, the H–C_C_ position of Cs[**Cl**_**12**_**-1**] surprisingly does not show any intermolecular interaction in the structure.

**Figure 4:**
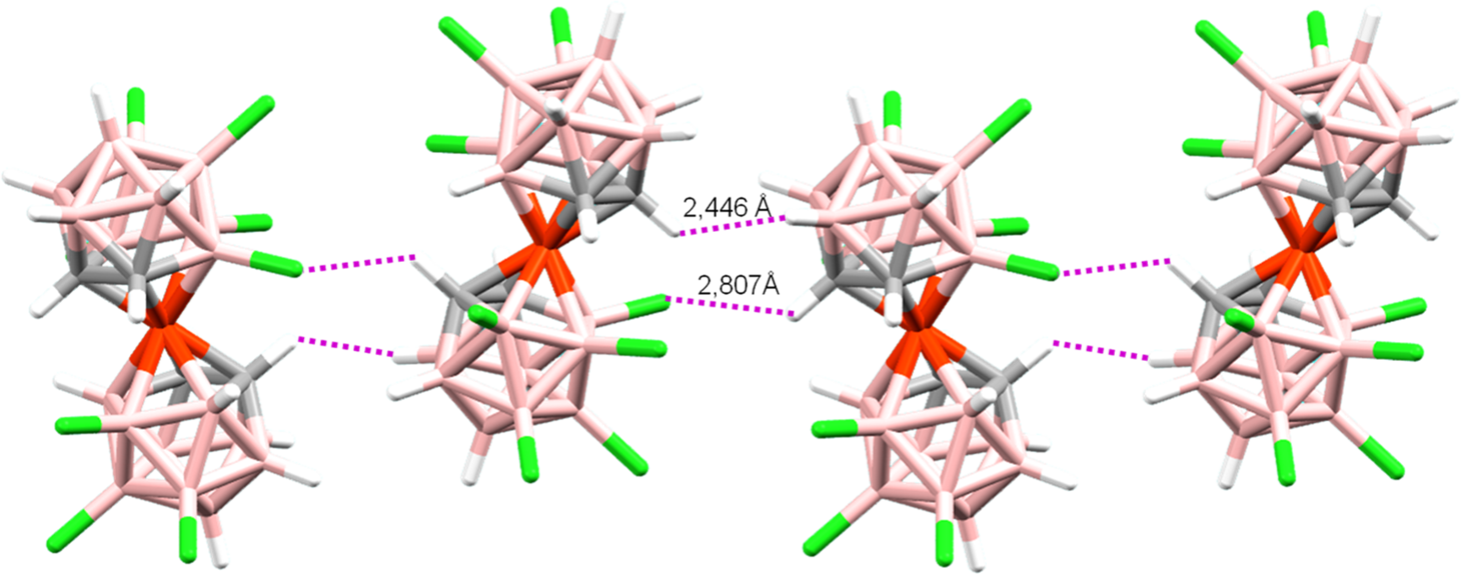
Interactions C_A_(2)–H···Cl–B_B_(4′) and B_A_(6)–H···H–C_B_(1) between near [**Cl**_**8**_**-1**]^−^ molecules, forming a chain in the crystal packing (B is in pink, C in gray, H in white, Cl in green, and Co^3+^ in orange).

As a result of all of these contacts, the three structures revealed the less common conformations in the cobaltabis(dicarbollide) derivatives.^66^ The crystal structure of [NMe_4_][**Cl**_**8**_**-1**] shows a transoid rotamer in the solid state, even though this conformation should be the least energetic and the intermolecular interactions and crystal packaging used are the key factors (Figure 4). However, in this structure, we have a solid solution; hence, the packaging should allow the exchange of chlorine and hydrogen and vice versa in the B(4) and B(4′) positions, and the trans conformation to obtain it is more advantageous.

**Figure 5:**
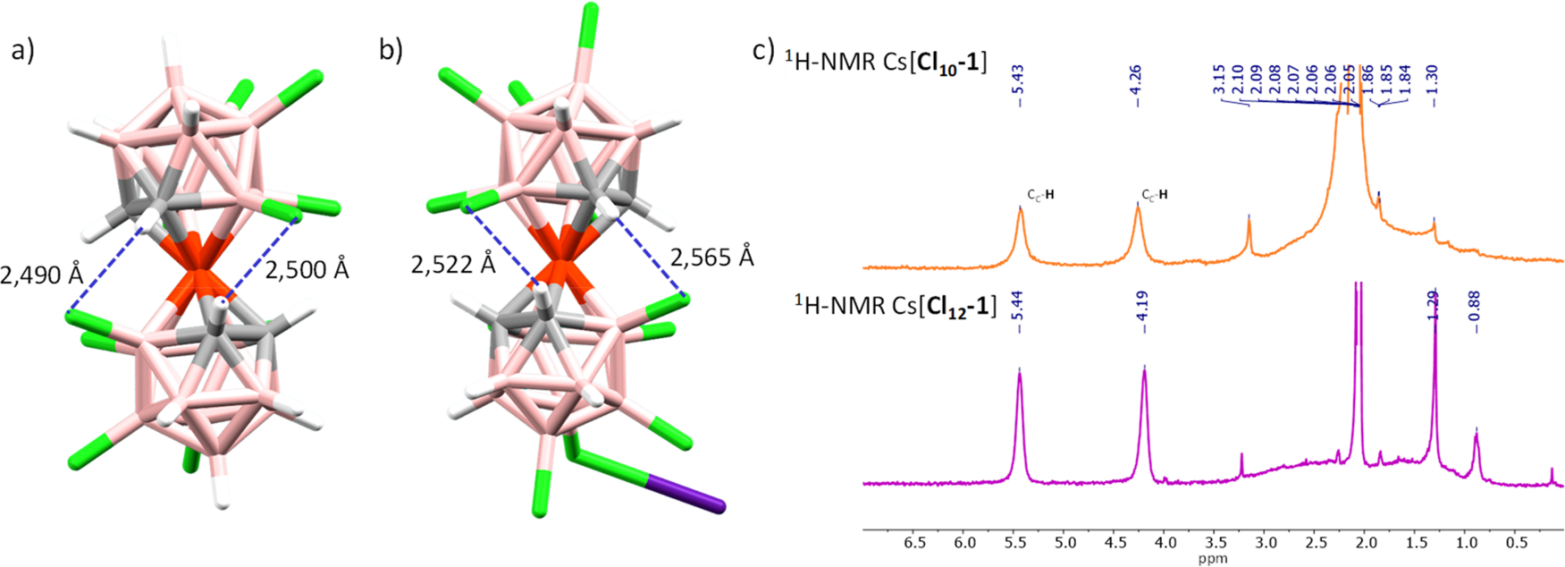
B(7)–Cl···H–C(1′) and C(2)–H···Cl–B(4′) intramolecular interactions of the structures (a) [**Cl**_**10**_**-1**]^−^ and (b) [**Cl**_**12**_**-1**]^−^, responsible for their gauche conformation. (c) ^1^H NMR spectra in (CD_3_)_2_CO of Cs[**Cl**_**10**_**-1**] (in orange) and Cs[**Cl**_**12**_**-1**] (in purple).

Structures [NMe_4_][**Cl**_**10**_**-1**] and Cs[**Cl**_**12**_**-1**] surprisingly present gauche 1 and gauche 2 rotamers with centroid distances between η^5^-C_2_B_3_ and Co^III^ of 1.514 and 1.539 Å, respectively, typical distances of this conformation (Figure 3).^66^ These conformations are not the most common or the least energetic. However, while the structure of [**Cl**_**8**_**-1**]^−^ presents distances of around 2.75 Å between atoms of two cluster cages of the same molecule, both crystal structures [NMe_4_][**Cl**_**10**_**-1**] and Cs[**Cl**_**12**_**-1**] present shorter intramolecular interactions that force the gauche conformation of the molecules, namely, the interactions B(7)–Cl···H–C(1′) and C(2)–H···Cl–B(4′) with distances of 2.490 and 2.500 Å, respectively, for the crystal structure of [NMe_4_][**Cl**_**10**_**-1**] and 2.522 and 2.565 Å, respectively, for the crystal structure of Cs[**Cl**_**12**_**-1**] (Figure 5a,b). In addition, these intermolecular interactions are so strong that they persist in solution, explaining the extra peaks that appears in the ^11^B{^1^H} NMR spectra of both compounds (see the SI). The gauche conformation in the crystal structures of [NMe_4_][**Cl**_**10**_**-1**] and Cs[**Cl**_**12**_**-1**] breaks all of the symmetry of the molecule and, consequently, the ^1^H NMR spectrum shows two C_C_–H resonances with 2:2 intensities because the two C_C_–H bonds are spectroscopically different (Figure 5c). Moreover, the signal downfield supports the fact that the intramolecular B–Cl···H–C_c_ interactions are kept in solution.

The ^11^B NMR spectrum of Cs[**Cl**_**10**_**-1**] displays three singlets at 12.4, 6.4, and 1.6 ppm with intensities 2:6:2, corresponding to 10 B–Cl units at B(8,8′), B(4,4′, 9,9′,12,12′), and B(7,7′). In addition, the four doublets that appear in the ^11^B NMR spectrum at 0.2, −14.3, −17.1, and −27.7 ppm, with intensities 2:2:2:2, correspond to the B–H units of the boron atoms B(10,10′), B(5,5′,11,11′), and B(6,6′), respectively. In contrast, the ^11^B NMR spectrum of Cs[**Cl**_**12**_**-1**] presents three singlets at 11.5, 5.6, and 0.7 ppm with intensities 2:8:2, corresponding to B(8,8′), B(4,4′,9,9′,10,10′,12,12′), and B(10,10′), respectively, which confirms the 12-boron-cluster vertex substitution. Two further doublets with intensities 4:2, corresponding to the B(5,5′,11,11′) and B(6,6′) B–H vertices, respectively, are also observed (see the SI).

Moreover, the ^11^B NMR spectra for Cs[**Cl**_**10**_**-1**] and Cs[**Cl**_**12**_**-1**] unveil a B(4)–Cl signal very different from its supposed equivalent B(7)–Cl (Δppm = 4.81), demonstrating again that the compounds retain the intramolecular B–Cl···H–C_c_ interactions in solution, as shown above by ^1^H NMR.

**Figure 6:**
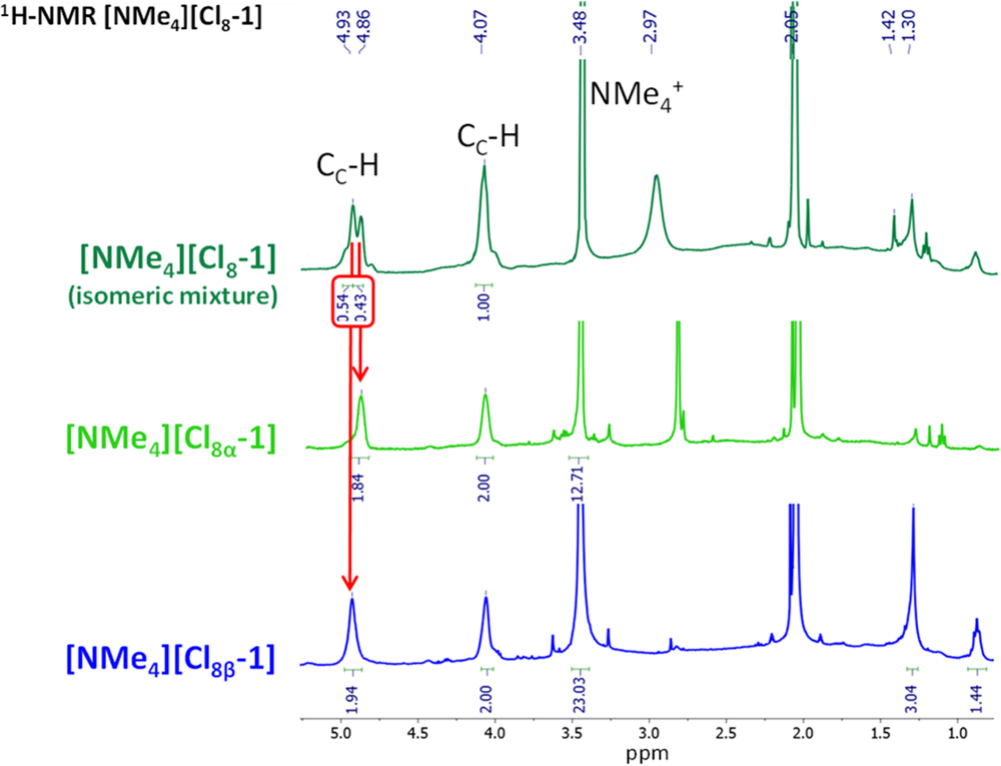
^1^H NMR spectra in (CD_3_)_2_CO of the mixture [NMe_4_][**Cl**_**8**_**-1**] (dark green), isolated [NMe_4_][**Cl**_**8α**_**-1**] (light green), and isolated [NMe_4_][**Cl**_**8β**_**-1**] (blue).

Concerning the NMR characterization of [NMe_4_][**Cl**_**8**_**-1**], the situation is very different (Figure 6). Even though the elemental analysis and MALDI-TOF-MS at the negative mode confirm the purity of the product, the ^11^B NMR spectrum shows that many signals are difficult to characterize because of the presence of the different structural isomers [**Cl**_**8α**_**-1**]^−^ and [**Cl**_**8β**_**-1**]^−^. The ^1^H NMR show two signals at 4.92 and 4.87 ppm and one broad signal at 4.07 ppm, indicating the existence of an isomeric mixture, but integration of the proton peaks corresponding to the C_c_–H signals of the ^1^H NMR spectrum provides a rough ratio of 55:45 α/β isomers (Figure 6). Fortunately, separation of the mixture was possible thanks to the different polarities of the isomers. In particular, the isomer [**Cl**_**8β**_**-1**]^−^ was very insoluble in chloroform, leading to an isomeric pure product that could be analyzed by ^1^H, ^11^B, and ^13^C{^1^H} NMR (see the SI).

### Electrochemical Redox Couples

**Figure 7:**
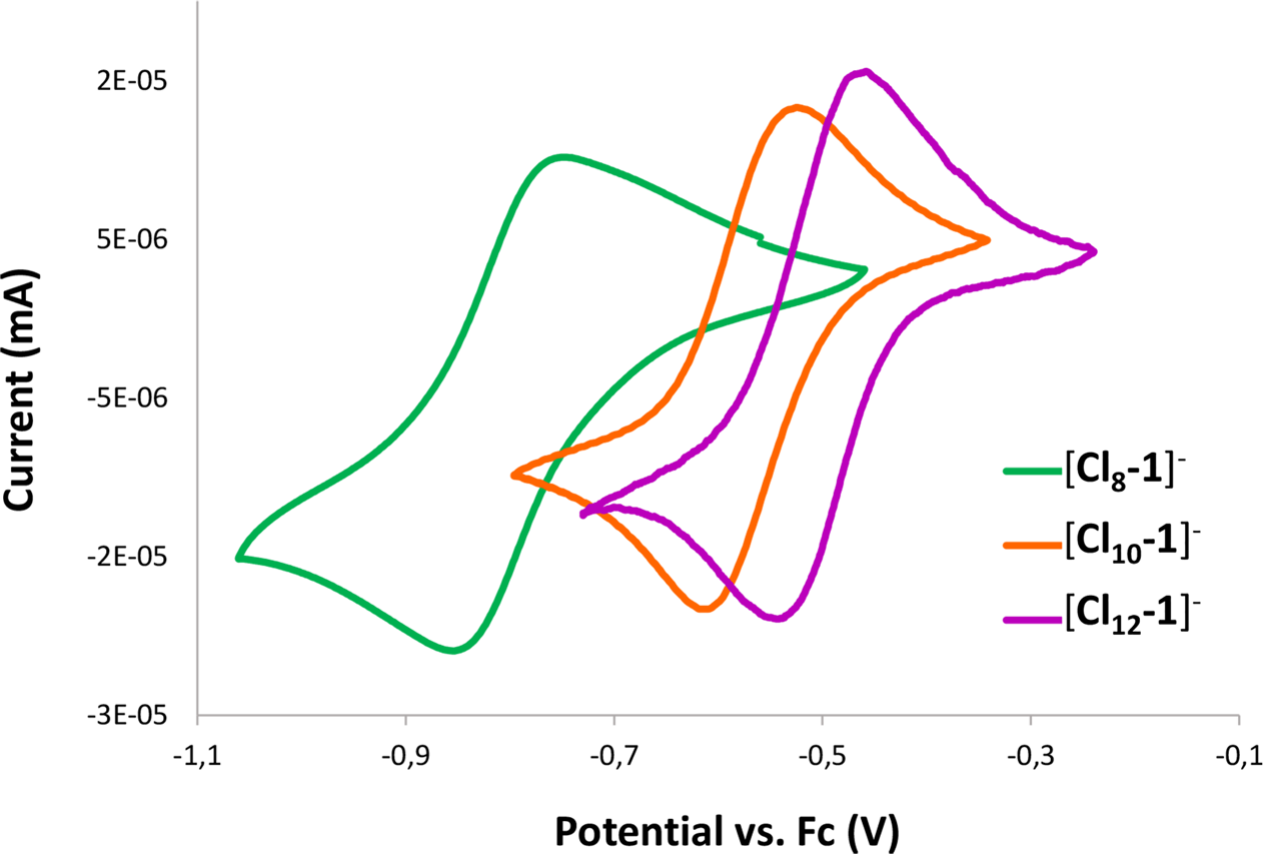
CV curves of [**Cl**_**8**_**-1**]^−^ (green), [**Cl**_**10**_**-1**]^−^ (orange), and [**Cl**_**12**_**-1**]^−^ (purple) carried out in dry acetonitrile as the solvent and [N*n*Bu_4_][PF_6_] (0.1M) as the supporting electrolyte. Glassy carbon was used as the working electrode, Ag as the pseudoreference electrode, and Pt wire as the counter electrode. Measurements were referenced to an internal Fc^+^/Fc couple.

**Figure 8:**
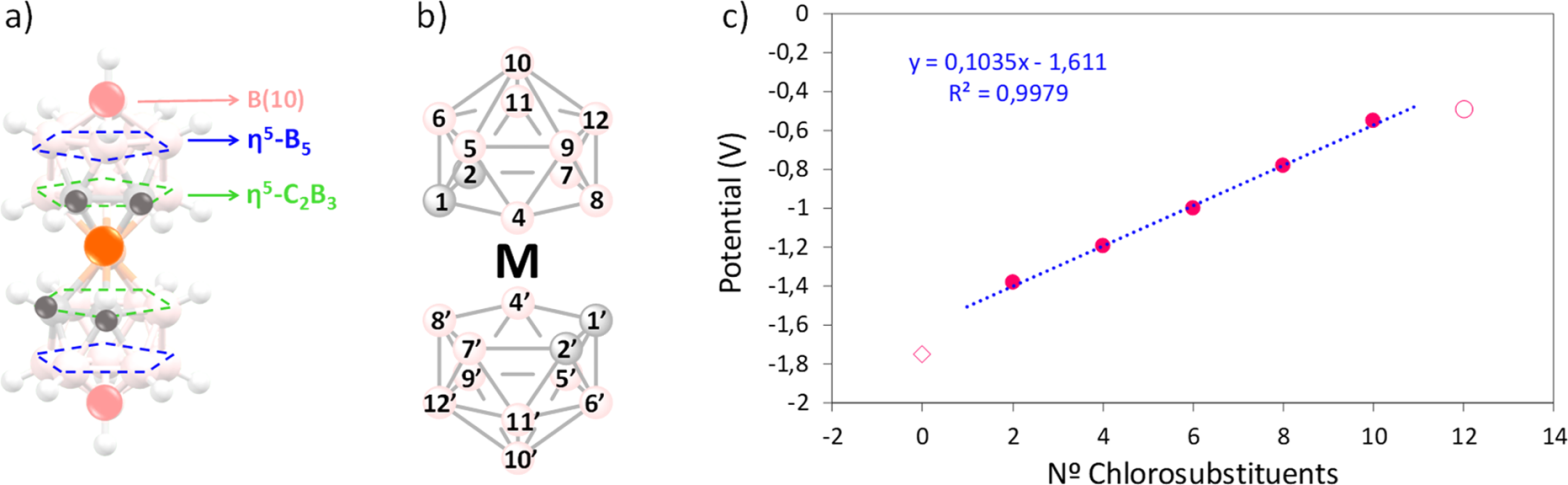
(a) Representation of the different planes of [**1**]^−^. (b) Scheme of [**1**]^−^ representing the vertex numbering. (c) Graphical representation of *E*_1/2_(Co^III^/Co^II^) (V) varying with the number of chloro substituents in the [**1**]^−^ structure.

The *E*_1/2_(Co^III^/Co^II^) values for [**Cl**_**8**_**-1**]^−^, [**Cl**_**10**_**-1**]^−^, and [**Cl**_**12**_**-1**]^−^ were experimentally obtained by cyclic voltammetry (CV) and compared with the other chlorinated derivatives available in the literature (Figures 7 and 8 and Table 1). These results indicated not only the redox potential of Co^III^/Co^II^ but also the reversibility of the system. For [NMe_4_][**Cl**_**10**_**-1**] and [NMe_4_][**Cl**_**12**_**-1**], their ΔmV values are less than 100, 99.8, and 62 mV. On the other hand, [NMe_4_][**Cl**_**8**_**-1**] shows a broader signal with a ΔmV of 183 mV (Figure 7 and Table 1), most likely due to the mixture of isomers, which causes slightly different potentials, and because of the overlap of the two traces, a thicker signal is found.

**Table 1:** *E*_1/2_(Co^III^/Co^II^) Data for [**Cl**_***n***_**-1**]^−^ (*n* = 0, 1, 2, 4, 6, 8, 10, and 12)^a^

compound	*E*1/2 versus Fc^+^/Fc (V) [ΔmV]	Δ*E*1/2 (V)
[**1**]^−^	–1.75 [56]^72^	
[**Cl**_**2**_**-1**]^−^	–1.39^73^	0.36
[**Cl**_**4**_**-1**]^−^	–1.20^62^	0.19
[**Cl**_**6**_**-1**]^−^	–1.01^63^	0.19
[**Cl**_**8**_**-1**]^−^	–0.79 [183]	0.22
[**Cl**_**10**_**-1**]^−^	–0.56 [100]	0.23
[**Cl**_**12**_**-1**]^−^	–0.49 [63]	0.07

a The ΔmV data indicate the potential difference between the reduction and oxidation peaks. Δ*E*_1/2_ is the redox potential difference between compounds [**Cl**_***n***_**-1**]^−^ and [**Cl**_**(*****n*****–2)**_**-1**]^−^.

As a rule of thumb, it was considered that each new chloro added to the structure contributes +0.12 V to the *E*_1/2_(Co^III^/Co^II^) value.^56^Figure 8 shows that indeed the increment of *E*_1/2_(Co^III^/Co^II^) (Δ*E*_1/2_) is quasilinear, except for the first ([**Cl**_**2**_**-1**]^−^) and last ([**Cl**_**12**_**-1**]^−^) points, showing a considerable deviation from the expected values. This accounts for the importance of the chlorinated position, a phenomenon previously observed in the iodinated derivatives.^68^ It has been demonstrated that the anionic [**1**]^−^ cluster is a global 3D aromatic system^69^ with a negative charge delocalized all over the system.^70^ Considering that the chloro substituent is an electron-withdrawing group, each additional chloride makes the redox site more positive, and consequently the redox potential of the couple Co^III^/Co^II^ becomes more positive and then easier to reduce. In addition, the impact of this effect depends on the distance of the chlorinated position to the cobalt atom. The substituents that are in the plane nearest to cobalt (η^5^-C_2_B_3_) affect the redox potential of the Co^III^/Co^II^ couple more than those on a more distant plane (B_5_) or in the B(10) position (Figure 8a). Theoretical studies^71^ and the crystal structures of [NMe_4_][**Cl**_**8**_**-1**], [NMe_4_][**Cl**_**10**_**-1**], and Cs[**Cl**_**12**_**-1**] suggest that the chlorination order for [**1**]^−^ is first B(8), followed by B(9,12) (equivalent positions), B(4,7), B(8), B(5,11), and finally B(6), where the last three are very difficult to chlorinate. Therefore, the largest potential gaps are found for [**Cl**_**2**_**-1**]^−^ and [**Cl**_**12**_**-1**]^−^, which correspond to the chlorination of B(8) and B(10), respectively. To demonstrate our hypothesis, we synthesized [NMe_4_][3,3′-Co(4,7-Cl_2_-1,2-C_2_B_9_H_9_)_2_] and studied its electrochemistry. The synthesis was done following the methodology already described with some minor modifications (see the SI for the synthesis and characterization of [NMe_4_][3,3′-Co(4,7-Cl_2_-1,2-C_2_B_9_H_9_)_2_]);^53^ notice the distinct positions of chlorination of [NMe_4_][3,3′-Co(4,7-Cl_2_-1,2-C_2_B_9_H_9_)_2_] with regard to what we call [**Cl**_**4**_**-1**]^−^, [NMe_4_][3,3′-Co(8,9-Cl_2_-1,2-C_2_B_9_H_9_)_2_]. In [**Cl**_**4**_**-1**]^−^, one B–Cl is in the plane η^5^-C_2_B_3_ next to cobalt and the second B–Cl is in the more distant plane B_5_. In [NMe_4_][3,3′-Co(4,7-Cl_2_-1,2-C_2_B_9_H_9_)_2_], both B–Cl bonds are in the plane next to cobalt. Thus, *E*_1/2_(Co^III^/Co^II^) should be more positive in [NMe_4_][3,3′-Co(4,7-Cl_2_-1,2-C_2_B_9_H_9_)_2_]. The CV experiment presents a redox potential of −1.13 V versus Fc^+^/Fc, a value of *E*_1/2_(Co^III^/Co^II^) = 0.07 V more positive than the −1.20 V for [**Cl**_**4**_**-1**]^−^ (Table 1). In addition, this experiment demostrates the hypothesis of a broad ΔmV value for [**Cl**_**8**_**-1**]^−^ due to overlap of the two redox curves for the two isomers [**Cl**_**8α**_**-1**]^−^ and [**Cl**_**8β**_**-1**]^−^, proving that both are reversible systems.

## Conclusions

In this paper, we have demonstrated that, with a single platform, [3,3′-Co-(1,2-C_2_B_9_H_11_)_2_], [**1**]^−^, with a restricted number of equivalent sites, it has been possible by sequential halogenation to chlorinate up to 12 out of 18 possible positions. Earlier work indicated that each chloro substitution results in a potential modulation in the range 0.1–0.13 V. However, as is demonstrated here, it depends on the distance of the substitution site to the metal center. As a rule of thumb, the number of chloro substituents × 0.1 is a quite predictive equation for the voltage modulation. We mentioned earlier that our system was quite simple. To do this, we synthesize the molecule with the desired potential in a single reaction in a single flask, and this was achieved. In this way, we have made derivatives with 8, 10, and 12 chloro substituents, [**Cl**_**8**_**-1**]^−^, [**Cl**_**10**_**-1**]^−^, and [**Cl**_**12**_**-1**]^−^, where [**1**]^−^ is [Co(C_2_B_9_H_11_)_2_]^−^. They add to the [**Cl-1**]^−^, [**Cl**_**2**_**-1**]^−^, [**Cl**_**4**_**-1**]^−^, and [**Cl**_**6**_**-1**]^−^ described earlier. A total of 1.3 V is modulated stepwise with the particularity that each molecule occupies the same or a very similar volume so that solid solutions have crystallographically been encountered. This is a major breakthrough, but we cannot go further with [**1**]^−^ because substitutions with more than 12 chloro substituents are very difficult, at least with the current procedure. Does this mean that we cannot go beyond these potential values with metallacarboranes? The answer is yes, it is possible, and this is what we are working on now. If instead of using only cobalt, we use the much more abundant iron, i.e., we move from [Co(C_2_B_9_H_11_)_2_]^−^ to [Fe(C_2_B_9_H_11_)_2_]^−^, we shift all at once 1 V to more positive values. It is only to be expected that the tuning achieved by halogenation is comparable to [**1**]^−^, and the first results support this. [Fe(C_2_B_9_H_11_)_2_]^−^ in terms of the potential is equivalent to [**Cl**_**8**_**-1**]^−^. We hope that, with only two metals and the same platform, a potential range equivalent to 2 V can be achieved.

Up to now, [**Cl**_**6**_**-1**]^−^ was the highest chloro derivative of COSAN synthetically quasi-pure. Now, after 39 years since the first synthesis of [**Cl**_**6**_**-1**]^−^, three new highly chlorinated derivatives of [**1**]^−^ are introduced in good yield to the group of chloro derivatives of [**1**]^−^: [**Cl**_**8**_**-1**]^−^, [**Cl**_**10**_**-1**]^−^, and [**Cl**_**12**_**-1**]^−^.

## Experimental Section

### Materials

SO_2_Cl_2_ and AlCl_3_ were purchased from Sigma-Aldrich. H[COSAN] was synthesized from Cs[COSAN], as previously described.

### Synthesis

#### Synthesis of [NMe_4_][**Cl**_**8**_**-1**] (Isomeric Mixture)

A total of 50 mg (0.15 mmol) of H[3,3′-Co-(1,2-C_2_B_9_H_11_)_2_] in 8 mL (49.5 mmol) of SO_2_Cl_2_ was heated in an Ace pressure tube at 70 °C for 4 days. When the reaction has finished, the closed tube was left cool at room temperature. The tube was opened, and the solvent was removed under reduced pressure. The solid was extracted with diethyl ether and 0.1 M HCl three times, and the organic layer was cleaned with water once more. The solvent of the organic layer was removed under reduced pressure, the solid was dissolved in 5 mL of H_2_O, and a saturated solution of NMe_4_·HCl was added, leading to the appearance of a red solid precipitate, which was identified as an isomeric mixture of NMe_4_[**Cl_8α_-1**] and NMe_4_[**Cl_8β_-1**]. The solid was filtered and dried, obtaining 64.5 mg of an orange solid (yield: 70%). The NMe_4_[**Cl_8β_-1**] isomer was isolated using chloroform as a cleaning solvent (the β isomer is completely insoluble in chloroform). FTIR (ν in cm^–1^): 3046.01 and 2923.56 (C–H), 2593.79 (B–H), 2360.44 and 2339.23 (B–Cl). MALDI-TOF-MS. Theor.: *m*/*z* 598.96. Found: *m*/*z* 598.72. Elem anal. Calcd for CsCl_8_CoC_4_B_18_H_14_: C, 6.56; H, 1.91. Found: C, 6.51; H, 1.97. NMR characterization of [NMe_4_][**Cl**_**8α**_**-1**]). ^1^H{^11^B} NMR (400 MHz, CD_3_COCD_3_): δ 4.93 (2H, s, C–H), 4.06 (2H, s, C–H), 3.45 (12H, s, N(CH_3_)_4_). ^11^B NMR (128 MHz, CD_3_COCD_3_): δ 12.8 (2B, s, B(8,8′)–Cl), 5.4 (8B, B(4,7,8,8′,9,9′,12,12′)–Cl and B(4′,7′)–H), −2.3 (1B, d, ^1^*J*_B–H_ = 125 Hz, B(10)–H), −3.2 (1B, d, ^1^*J*_B–H_ = 122.9 Hz, B(10′)–H), −17.5 (2B, d, ^1^*J*_B–H_ = 166.4 Hz, B(5,11)–H), −19.7 (2B, d, ^1^*J*_B–H_ = 174.1 Hz, B(5′,11′)–H), −24.4 (1B, d, ^1^*J*_B–H_ = 151.0 Hz, B(6)–H), −27.2 (1B, d, ^1^*J*_B–H_ = 193.3 Hz, B(6′)–H). ^13^C{^1^H} NMR (100 MHz, CD_3_COCD_3_): δ 55.25. NMR characterization of [NMe_4_][**Cl**_**8β**_**-1**].^1^H{^11^B} NMR (400 MHz, CD_3_COCD_3_): δ 4.88 (2H, s, C–H), 4.08 (2H, s, C–H), 3.45 (12H, s, N(CH_3_)_4_). ^11^B NMR (128 MHz, CD_3_COCD_3_): δ 9.1 (2B, s, B(8)–Cl), 4.4 (2B, s, B(7)–Cl), 3.0 (4B, s, B(9,12)–Cl), −0.2 (2B, d, ^1^*J*_B–H_ = 153.6 Hz, B(4)–H), −4.0 (2B, d, ^1^*J*_B–H_ = 169.0 Hz, B(10)–H), −17.5 (2B, d, ^1^*J*_B–H_ = 163.6 Hz, B(11)–H), −21.9 (2B, d, ^1^*J*_B–H_ = 165.2 Hz, B(5)–H), −25.8 (2B, d, ^1^*J*_B–H_ = 169.0 Hz, B(6)–H). ^13^C{^1^H} NMR (100 MHz, CD_3_COCD_3_): δ 55.21

#### Synthesis of [NMe_4_][3,3′-Co-(4,7,8,9,12-Cl_5_-1,2-C_2_B_9_H_6_)_2_]

A mixture of 500 mg (1.54 mmol) of H[3,3′-Co-(1,2-closo-C_2_B_9_H_11_)_2_], 20.5 mg (0.15 mmol) of AlCl_3_, and 8 mL (99 mmol) of SO_2_Cl_2_ was heated in an Ace pressure tube at 70 °C for 2 days. When the reaction has finished, the closed tube was left to cool at room temperature. The tube was opened, and the solvent was removed under reduced pressure. A total of 20.5 mg (0.15 mmol) of AlCl_3_ was added again, and the mixture was dissolved in 8 mL of SO_2_Cl_2_ in the same Ace pressure tube. The reaction was heated at 70 °C for another 2 days. When the reaction had finished, the closed tube was left to cool at room temperature. The product was purified following the same treatments as those used with the compound [NMe_4_][**Cl**_**8**_**-1**]. The solid was filtered and dried, and 924 mg of a red solid corresponding to the product NMe_4_[**Cl_10_-1**] was obtained (yield: 90%). ^1^H{^11^B} NMR (300 MHz, CD_3_COCD_3_): δ 5.43 (2H, s, C_Cluster_–H), 4.26 (2H, s, C_Cluster_–H). ^11^B NMR (96.3 MHz, CD_3_COCD_3_): δ 12.4 (2B, s, B(8)–Cl), 6.4 (6B, s, B(4)–Cl, B(9)–Cl, or B(12)–Cl), 1.6 (2B, s, B(7)–Cl), 0.2 (2B, d, ^1^*J*_B–H_ = 142.1 Hz, B(10)–H), −14.3 (2B, d, ^1^*J*_B–H_ = 173.3 Hz, B–H), −17.1 (2B, d, ^1^*J*_B–H_ = 183.0 Hz, B–H), 27.7 (2B, d, ^1^*J*_B–H_ = 182.0 Hz, B(6)–H). ^13^C{H} NMR (75.5 MHz, CD_3_COCD_3_): δ 50.12(C_Cluster_–H), 48.12 (C_Cluster_–H). FTIR (ν in cm^–1^): 3059.51 and 3.037.34 (C–H), 2601.5 (B–H), 2360.4 and 2339.23 (B–Cl). MALDI-TOF-MS. Theor.: *m*/*z* 666.88. Found: *m*/*z* 666.75. Elem anal. Calcd for CsCl_10_CoC_4_B_18_H_12_: C, 5.99; H, 1.49. Found: C, 5.92; H, 1.56.

#### Synthesis of [NMe_4_][3,3′-Co-(4,7,8,9,10,12-Cl_6_-1,2-C_2_B_9_H_5_)_2_]

The mixture of 50 mg (0.15 mmol) of H[3,3′-Co-(1,2-C_2_B_9_H_11_)_2_], 20.5 mg (0.15 mmol) of AlCl_3_, and 8 mL (99 mmol) of SO_2_Cl_2_ was heated in an Ace pressure tube at 70 °C for 4 days. When the reaction had finished, the closed tube was left to cool at room temperature. The product was purified following the same treatments as those used with the compound [NMe_4_][**Cl**_**8**_**-1**]. The solid was filtered and dried, and 106 mg of a red solid corresponding to the product was obtained (yield: 94%). ^1^H{^11^B} NMR (300 MHz, CD_3_COCD_3_): δ 5.44 (2H, s, C_Cluster_–H), 4.19 (2H, s, C_Cluster_–H), 2.34 (4H, s, B(5)–H or B(11)–H). ^11^B NMR (96.3 MHz, CD_3_COCD_3_): δ 11.5 (2B, s, B(8)–Cl), 5.5 (8B, s, B(4)–Cl, B(7)–Cl, B(9)–Cl, B(12)–Cl), 0.8 (2B, s, B(10)–Cl), 14.2 (2B, d, ^1^*J*_B–H_ = 173.3 Hz, B(5)–H), 17.05 (2B, d, ^1^*J*_B–H_ = 183.0 Hz, B(11)–H), 27.93 (2B, d, ^1^*J*_B–H_ = 173.3 Hz, B(6)–H). ^13^C{^1^H} NMR (75.5 MHz, CD_3_COCD_3_): δ 48.18 (C_Cluster_–H), 46.28 (C_Cluster_–H). FTIR (ν in cm^–1^): 3060.48 and 2866.67 (C–H), 2591.86 (B–H), 2360.44 and 2339.23 (B–Cl). MALDI-TOF-MS. Theor.: *m*/*z* 734.81. Found: *m*/*z* 735.71. Elem anal. Calcd for NaCl_12_CoC_4_B_18_H_10_·CH_3_COOH: C, 8.77; H, 1.70. Found: C, 8.9; H, 1.94.

## Supporting Information Available

Instrumentation, materials and methods, syntheses and characterization of the compounds, ^11^B{^1^H}, ^1^H, ^1^H{^11^B}, ^11^B, and ^13^C{^1^H} NMR, MALDI-TOF-MS, IR, and CV, crystal structures, crystal packaging across the *a*, *b*, and *c* axes, and crystallographic data (PDF)
