# Supplementary material for: 1.3 V Inorganic Sequential Redox Chain with an All-Anionic Couple 1–/2– in a Single Framework
Source: Inorg Chem. 2021 Oct 24;60(21):16168–77. doi: 10.1021/acs.inorgchem.1c01822 (PMC9180739; doi:10.1021/acs.inorgchem.1c01822)
Supplement: Supplementary file 1 — ic1c01822_si_001.pdf [file ic1c01822_si_001.pdf]

SUPPORTING INFORMATION

1.3 Volts Inorganic Sequential Redox Chain with an all Anionic Couple 1-/2- in a Single Framework

Ana B. Buades,[a] Clara Viñas,[a] Xavier Fontrodona[b] and Francesc Teixidor[a,*]

[a] Institut de Ciència de Materials de Barcelona (ICMAB-CSIC), Campus UAB, 08193 Bellaterra, Spain.

[b] Dept. de Química and Serveis Tècnics de Recerca, Universitat de Girona, Campus de Montilivi, 17071 Girona, Spain.

Corresponding autor’s email: teixidor@icmab.es

INDEX

1. Instrumentation. 4

2. Materials and methods. 4

2.1. Synthesis of the cesium, tetramethylammonium, sodium and proton salts of the compound $[Cl_8\text{-}1]^-$, $[Cl_{10}\text{-}1]^-$ and $[Cl_{12}\text{-}1]^-$. 5

2.2. Characterization of $[NMe_4][3,3'\text{-}Co(8,9,12\text{-}Cl_3\text{-}1,2\text{-}C_2B_9H_8)(4,7,8,9,12\text{-}Cl_5\text{-}1,2\text{-}C_2B_9H_6)]$ ($Cs[Cl_{8\alpha}\text{-}1]$) and $[NMe_4][3,3'\text{-}Co(7,8,9,12\text{-}Cl_4\text{-}1,2\text{-}C_2B_9H_7)_2]$ ($Cs[Cl_{8\beta}\text{-}1]$) 6

Figure S1. $^1H\{^{11}B\}$-NMR of $[NMe_4]$[**Cl_8-1**]..6
Figure S2. ^{11}B-NMR of $[NMe_4]$[**Cl_8-1**]..6
Figure S3. $^{11}B\{^1H\}$-NMR of $[NMe_4]$[**$Cl_{8\alpha}$-1**]..7
Figure S4. $^{11}B\{^1H\}$-NMR of $[NMe_4]$[**$Cl_{8\beta}$-1**]..7
Figure S5. $^{13}C\{^1H\}$-NMR of $[NMe_4]$[**Cl_8-1**]..8
Figure S6. MALDI-TOF-MS of $[NMe_4]$[**Cl_8-1**]..8
Figure S7. IR spectrum of $[NMe_4]$[**Cl_8-1**]..9
Figure S8. Cyclic voltammetry of compound $[NMe_4]$[**Cl_8-1**]..9
Figure S9. Crystal structure of $[NMe_4]$[**Cl_8-1]**..9
Figure S10. Crystal packaging across the a-axis.. 10
Figure S11 Crystal packaging across the b-axis..10
Figure S12. Crystal packaging across the c-axis..10

2.3. Characterization of $Cs[3,3'\text{-}Co(4,7,8,9,12\text{-}Cl_5\text{-}1,2\text{-}C_2B_9H_6)_2]$ ($Cs[Cl_5\text{-}1]$). 111

Figure S13. ^1H-NMR of Cs[**Cl_{10}-1**]..11
Figure S14. $^1H\{^{11}B\}$-NMR of Cs[**Cl_{10}-1**]..11
Figure S15. ^{11}B-NMR of Cs[**Cl_{10}-1**]..12
Figure S16. $^{11}B\{^1H\}$-NMR of Cs[**Cl_{10}-1**]..12
Figure S17. $^{13}C\{^1H\}$-NMR of Cs[**C_{10}-1**]..13
Figure S18. MALDI-TOF-MS of Cs[**Cl_{10}-1**].. 13
Figure S19. IR spectrum of Cs[**Cl_{10}-1**].. 14
Figure S20. Cyclic voltammetry of $[NMe_4]$[**Cl_{10}-1**]..14
Figure S21. Representation of the crystalline structure $[NMe_4]$[**Cl_{10}-1**]. 14
Figure S22. Crystal packaging across the a-axis..15
Figure S23. Crystal packaging across the b-axis..15
Figure S24. Crystal packaging across the c-axis..15

2.4. Characterization of $Cs[3,3'\text{-}Co(4,7,8,9,10,12\text{-}Cl_6\text{-}1,2\text{-}C_2B_9H_5)_2]$ ($Cs[Cl_{12}\text{-}1]$). 16

Figure S25. ^1H-NMR of Cs[**Cl_{12}-1**]..16
Figure S26. $^1H\{^{11}B\}$-NMR of Cs[**Cl_{12}-1**]..16
Figure S27. ^{11}B-NMR of Cs[**Cl_{12}-1**]..17
Figure S28. $^{11}B\{^1H\}$-NMR of Cs[**Cl_{12}-1**]..17
Figure S29. $^{13}C\{^1H\}$-NMR of Cs[**Cl_{12}-1**]..18
Figure S30. MALDI-TOF-MS of Cs[**Cl_{12}-1**]..18

Figure S31. IR spectrum of Cs[**Cl_{12}-1**]......18
Figure S32. Cyclic voltammetry of Cs[**Cl_{12}-1**]......19
Figure S33. Crystalline structure of Cs[**Cl_{12}-1**]......19
Figure S34. Crystal packaging across the a-axis......20
Figure S35. Crystal packaging across the b-axis......20
Figure 36. Crystal packaging across the c-axis......20

2.5. Synthesis and characterization of [NMe_4][3,3'-Co-(4,7-Cl_2-1,2-$C_2B_9H_9$)$_2$]......21

Scheme S1......21
Figure S37. MALDI-TOF-MS of[NMe_4][3,3'-Co-(4,7-Cl_2-1,2-$C_2B_9H_9$)$_2$]......21
Figure S38. Cyclic voltammetry of [NMe_4][3,3'-Co-(4,7-Cl_2-1,2-$C_2B_9H_9$)$_2$]......22

3. Crystallographic data......22

4. References......23

1. Instrumentation.

NMR measurements: The ^{1}H NMR (400 MHz), ^{11}B NMR and $^{11}B\{^{1}H\}$ NMR (128.38 MHz), and $^{13}C\{^{1}H\}$ NMR (100 MHz) spectra were recorded with a Bruker Advance III (400MHz) instrument equipped with the appropriate decoupling accessories. All NMR spectra were performed in deuterated acetone (purchased from Sigma-Aldrich) at 22°C. ^{11}B NMR and $^{11}B\{^{1}H\}$ NMR resonances were referenced to external $BF_3{\cdot}OEt_2$, while $^{13}C\{^{1}H\}$ NMR shifts were referenced to $SiMe_4$. Chemical shifts are reported in units of parts per million downfield from reference, and all coupling constants in Hz.

MALDI-TOF Mass Spectra were collected in the negative mode using a Brucker Biflex instrument (N_2 laser; λ_{exc} 337 nm, pulses of 0.5 ns), with an ion source of 20000 kV (Uis1) and 17500 kV (Uis2).

IR spectra were obtained on a PerkinElmer® Universal ATR Accessory spectrophotometer.

Elemental Analyses were performed using a Carlo Erbo EA 1108 microanalyser.

Cyclic Voltammetries were obtained with an Autolab PGSTAT 30 potentiostat from Eco-Chemie at a scan rate of 100 mV/s. A three-electrode set up was used, being a glassy carbon as the working electrode; an Ag/AgCl/TBACl (0.1M) as the reference electrode and a Pt wire as the counter electrode. All measurements were done in dry and pure acetonitrile with $TBAPF_6$ 0.1 M as the inert electrolyte. The concentrations of all the measured samples were always 1 mM. All solvents and electrolytes used for the electrochemical measurements were purchased from Sigma-Aldrich. *Reagent grade acetonitrile was pre-dried over $CaCO_3$, and then distilled over P_2O_5. Prior to use, acetonitrile was degassed by the standard freeze-pump-thaw technique in order to remove the dissolved oxygen, and stored over 0.4 nm molecular sieves. $TBAPF_6$ was dried overnight at 50º under vacuum to remove possible traces of water.

2. Materials and methods.

Cesium salt of cobaltabisdicarbollide was purchased from Katchem. The $[NMe_4]$[*nido*-9,11-Cl_2-7,8-$C_2B_9H_{10}$] started compound was synthesized following the literature method.[1] The protonated salt of $[3,3'\text{-Co-}(1,2\text{-}C_2B_9H_{11})_2]^-$ were synthesized according to an extraction procedure in diethyl ether as described.[2] Sulphuryl chloride, Aluminum chloride, cesium chloride and tetramethylammonium were purchased from Sigma-Aldrich and used as received.

X-ray Structure Determination. The crystals of $[NMe_4]$[**Cl_8-1**], $[NMe_4]$[**Cl_{10}-1**] and Cs[**Cl_{12}-1**] were immersed in cryo-oil, mounted in a MiTeGen loop, and measured at 274 K on a D8 QUEST ECO three-circle diffractometer equipped with a Ceramic x-ray tube, a doubly curved silicon crystal Bruker Triumph monochromator and using Mo Kα (λ = 0.71076 Å) radiation. The structures were solved and refined using the Bruker SHELXTL Software Package.[3] Data were corrected for absorption effects using the Multi-Scan method (SADABS[4]).

2.1. Synthesis of the cesium, tetramethylammonium, sodium and proton salts of the compound $[Cl_8\text{-}1]^-$, $[Cl_{10}\text{-}1]^-$ and $[Cl_{12}\text{-}1]^-$.

i) Synthesis of the cesium salt.

The $[NMe_4]$[$\mathbf{Cl_x}$**-1**] (x = 8, 10 or 12) is dissolved in diethyl ether and extract three times with HCl 0.1M water solution. The organic layer is evaporated with reduced pressure and the product is dissolved in water. Then a saturated water solution of cesium chloride (CsCl) is added since all product was precipitated.

ii) Synthesis of the tetramethylammonium salt.

The Cs[$\mathbf{Cl_x}$**-1**] (x = 8, 10 or 12) is dissolved in diethyl ether and extract three times with HCl 0.1M water solution. The organic layer is evaporated with reduced pressure and the product is dissolved in water. Then, a saturated water solution of tetramethylammonium chloride (NMe_4Cl) is added since all product was precipitated.

iii) Synthesis of the proton salt.

The $[NMe_4]$[$\mathbf{Cl_x}$**-1**] (x = 8, 10 or 12) is dissolved in diethyl ether and extract three times with HCl 0.1M water solution. The organic layer is dried with anhydrous Mg_2SO_4 and evaporated with reduce pressure.

iv) Synthesis of the sodium salt.

The $[NMe_4]$[$\mathbf{Cl_x}$**-1**] (x = 8, 10 or 12) is dissolved in diethyl ether. The organic solution was extract three times with HCl 0.1M water solution, three times with a NaOH 1M water solution and finally, once more with water. The organic layer is dried with anhydrous Mg_2SO_4 and evaporated with reduce pressure.

2.2. Characterization of [NMe_4][3,3'-Co(8,9,12-Cl_3-1,2-$C_2B_9H_8$)(4,7,8,9,12-Cl_5-1,2-$C_2B_9H_6$)] (Cs[$Cl_{8\alpha}$-1]) and [NMe_4][3,3'-Co(7,8,9,12-Cl_4-1,2-$C_2B_9H_7$)$_2$] (Cs[$Cl_{8\beta}$-1])

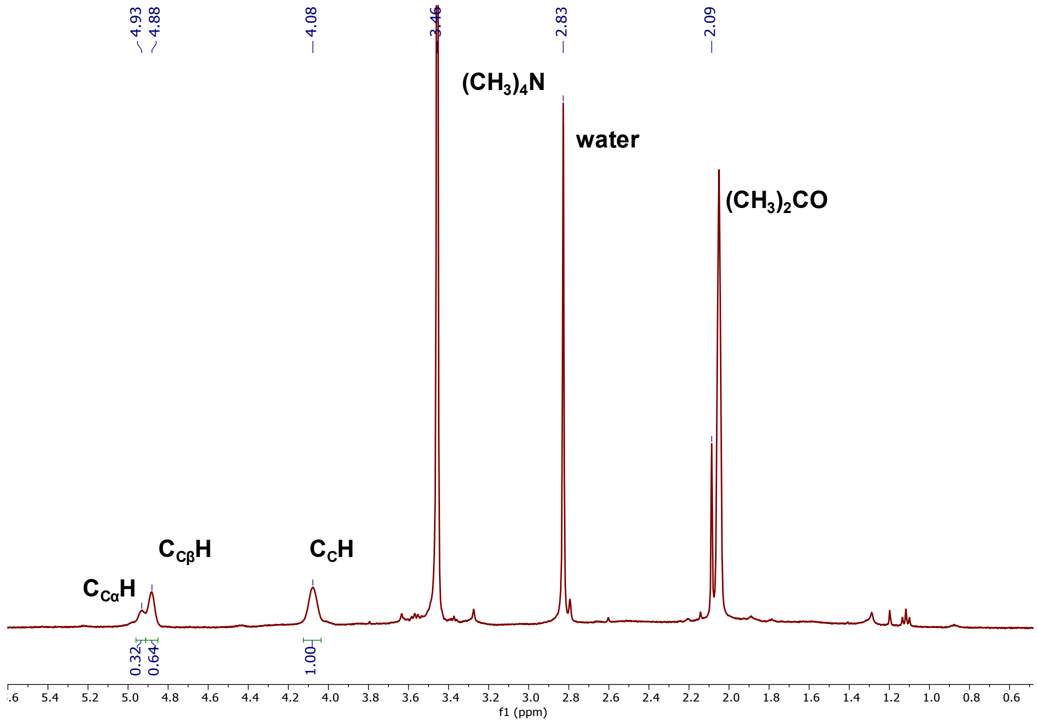

Figure S1. $^{1}H\{^{11}B\}$-NMR of [NMe_4][**Cl_8-1**].

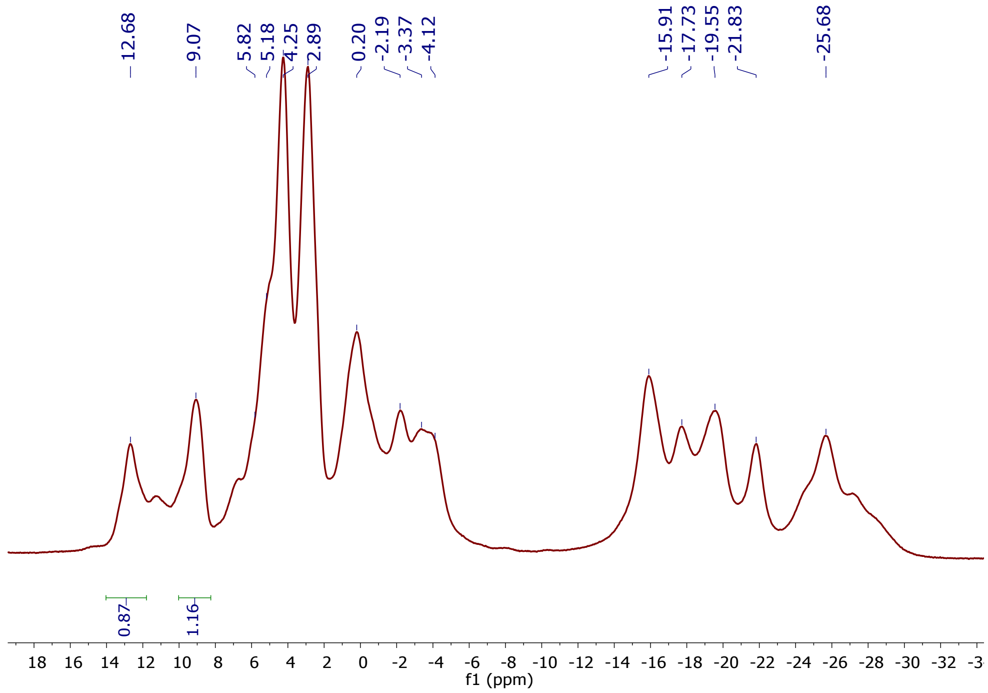

Figure S2. ^{11}B-NMR of [NMe_4][**Cl_8-1**].

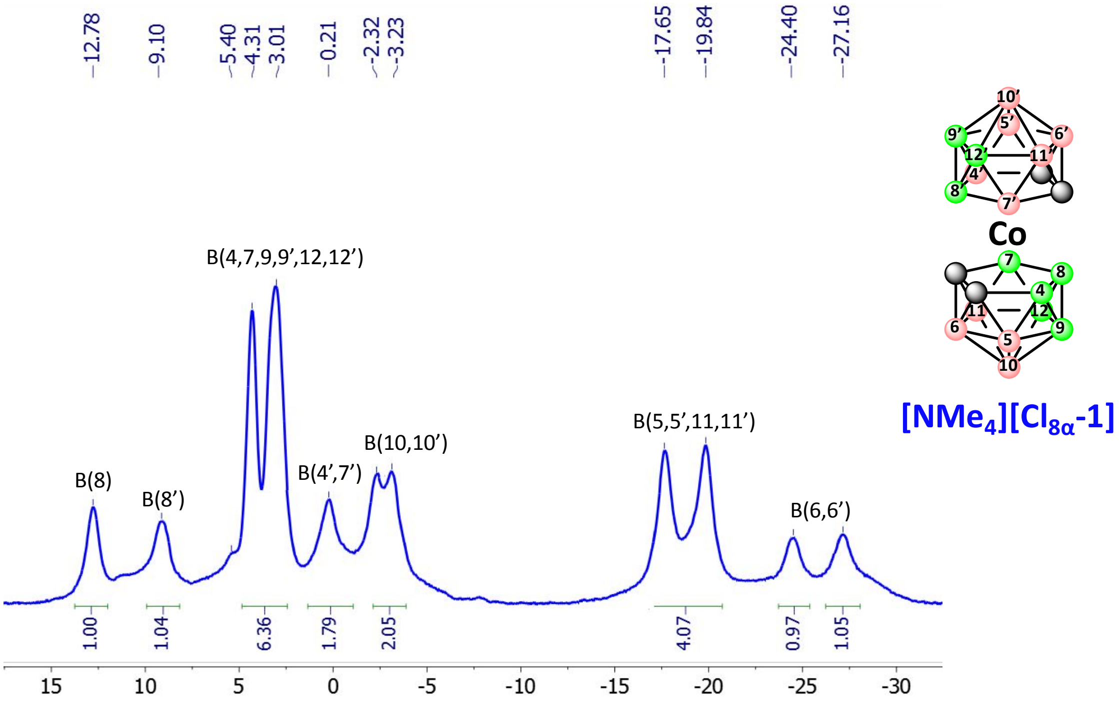

Figure S3. $^{11}B\{^{1}H\}$-NMR of [NMe_4][$\mathbf{Cl_{8\alpha}}$**-1**].

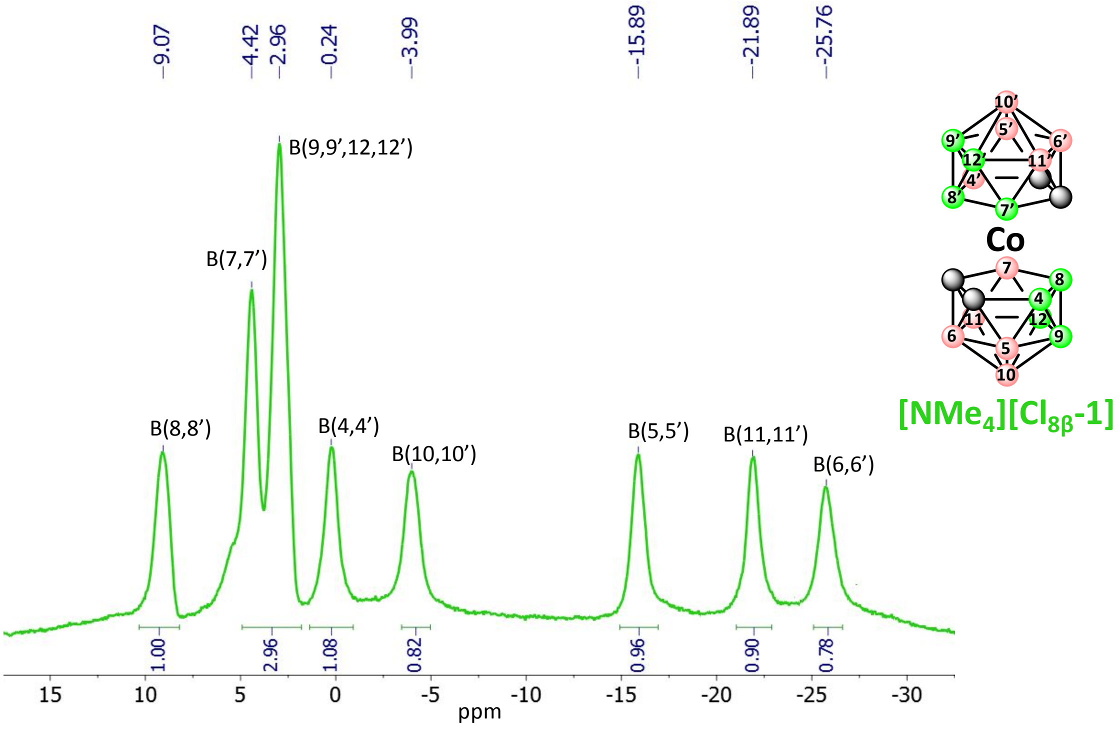

Figure S4. $^{11}B\{^{1}H\}$-NMR of [NMe_4][$\mathbf{Cl_{8\beta}}$**-1**].

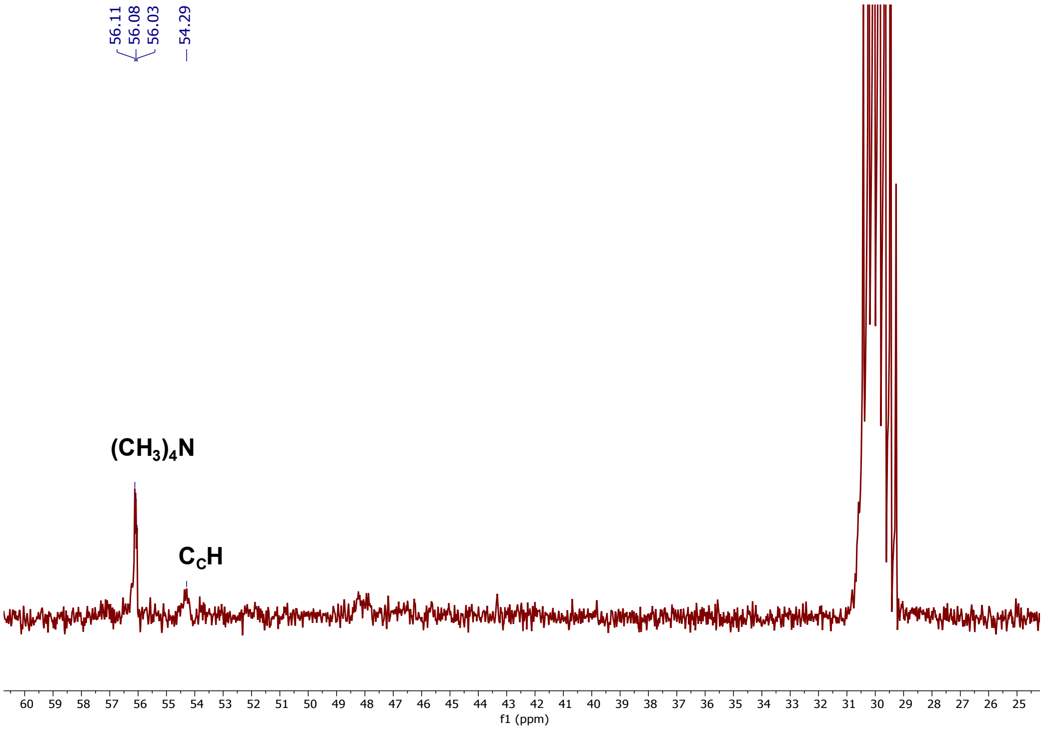

Figure S5. $^{13}C\{^1H\}$-NMR of $[NMe_4]$[$\mathbf{Cl_8}$-**1**].

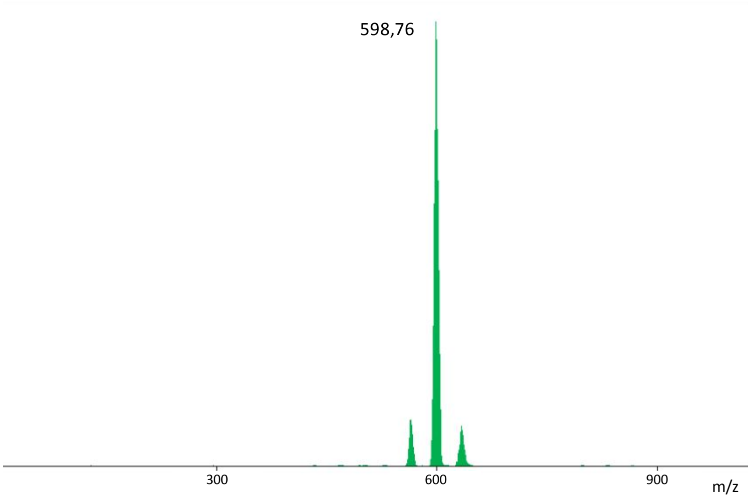

Figure S6. MALDI-TOF-MS of $[NMe_4]$[$\mathbf{Cl_8}$-**1**].

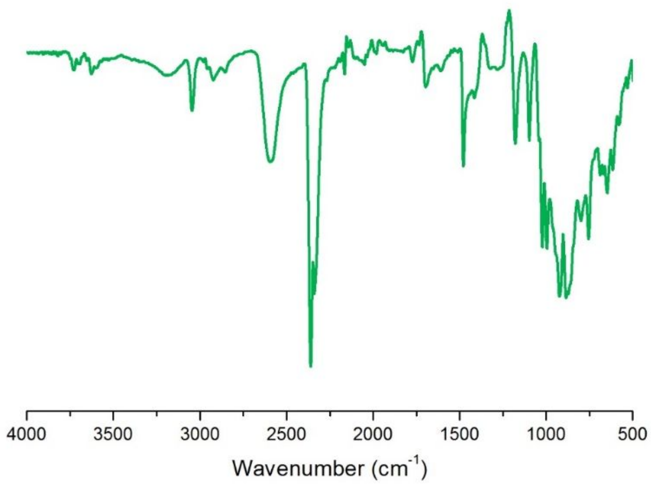

Figure S7. IR spectrum of [NMe_4][**Cl_8-1**].

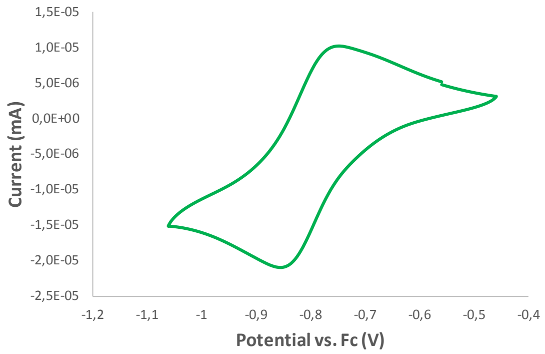

Figure S8. Cyclic voltammetry of compound [NMe_4][**Cl_8-1**]

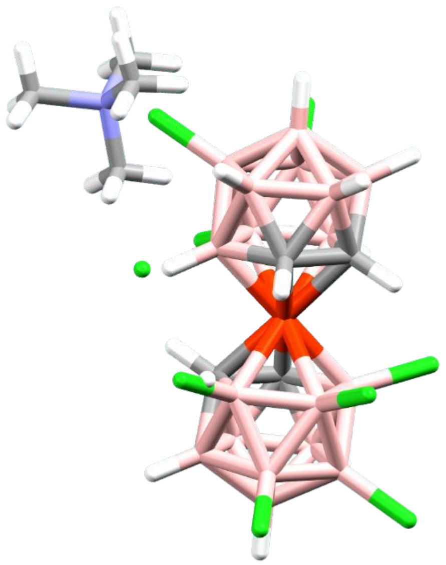

Figure S9. Crystal structure of [NMe_4][**Cl_8-1**], a the solid solution with a percentage of [NMe_4][**$Cl_{8\alpha}$-1**] (Cl3/Cl5) of 80% and [NMe_4][**$Cl_{8\beta}$-1**] (Cl4/Cl4) of 20%. An orange prism-like specimen of $C_8H_{26}B_{18}Cl_8CoN$, approximate dimensions 0.020 mm x 0.020 mm x 0.270 mm, was used for the X-ray crystallographic analysis. The crystal presents a P 21 21 21 space group with an orthorhombic crystallization system. Dimensions (Å) of the unit cell are ; **a** 12.791, **b** 14.768, **c** 15.060; with angles (º): **α** = **β** = **γ** = 90. The total volume of the unit cell is 2845 Å^3. The R value of the crystal is 4.62%.

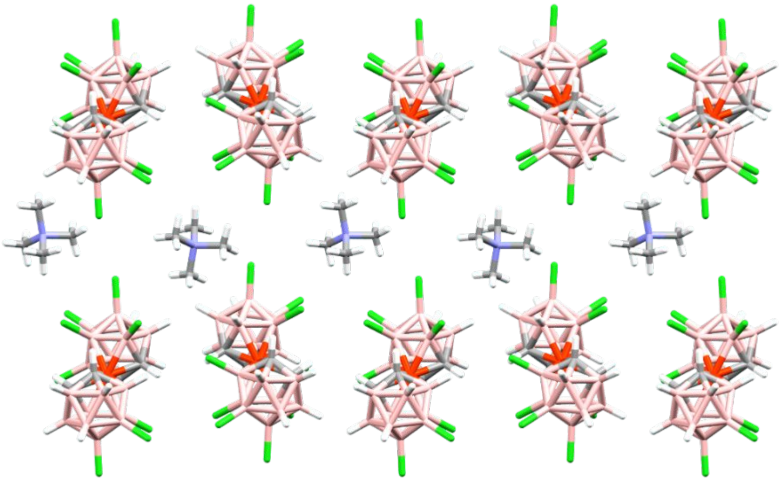

Figure S10. Crystal packaging across the a-axis

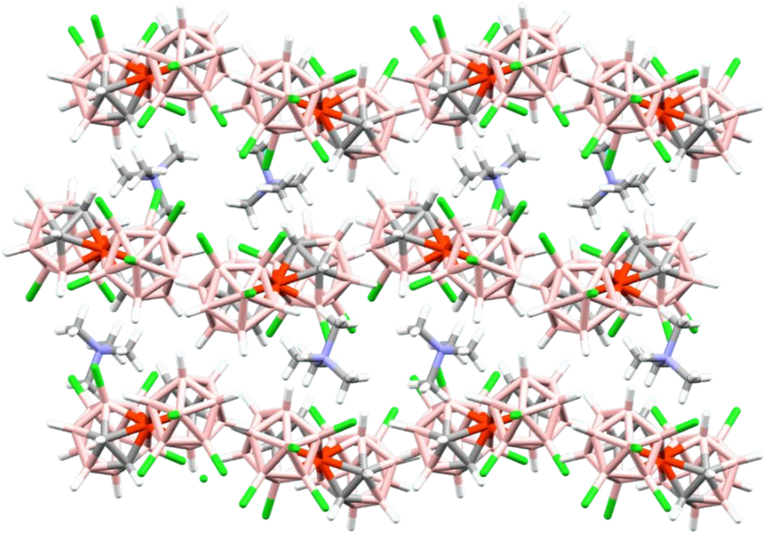

Figure S11 Crystal packaging across the b-axis

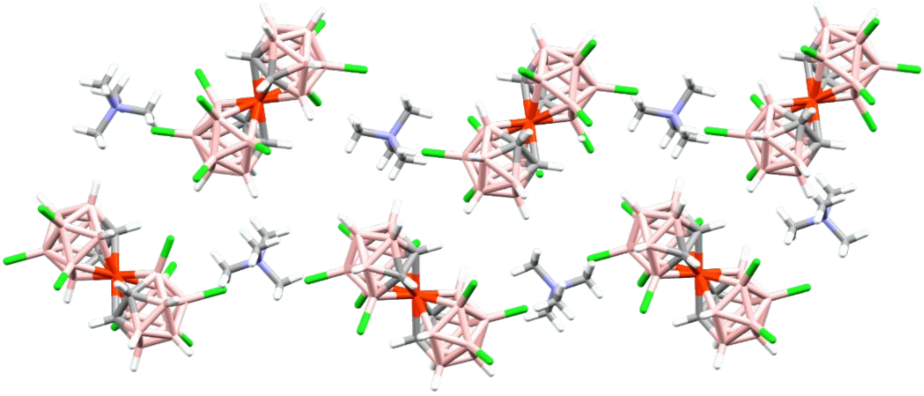

Figure S12. Crystal packaging across the c-axis

2.3. Characterization of Cs[3,3'-Co(4,7,8,9,12-Cl_5-1,2-$C_2B_9H_6$)$_2$] (Cs[Cl_5-1]).

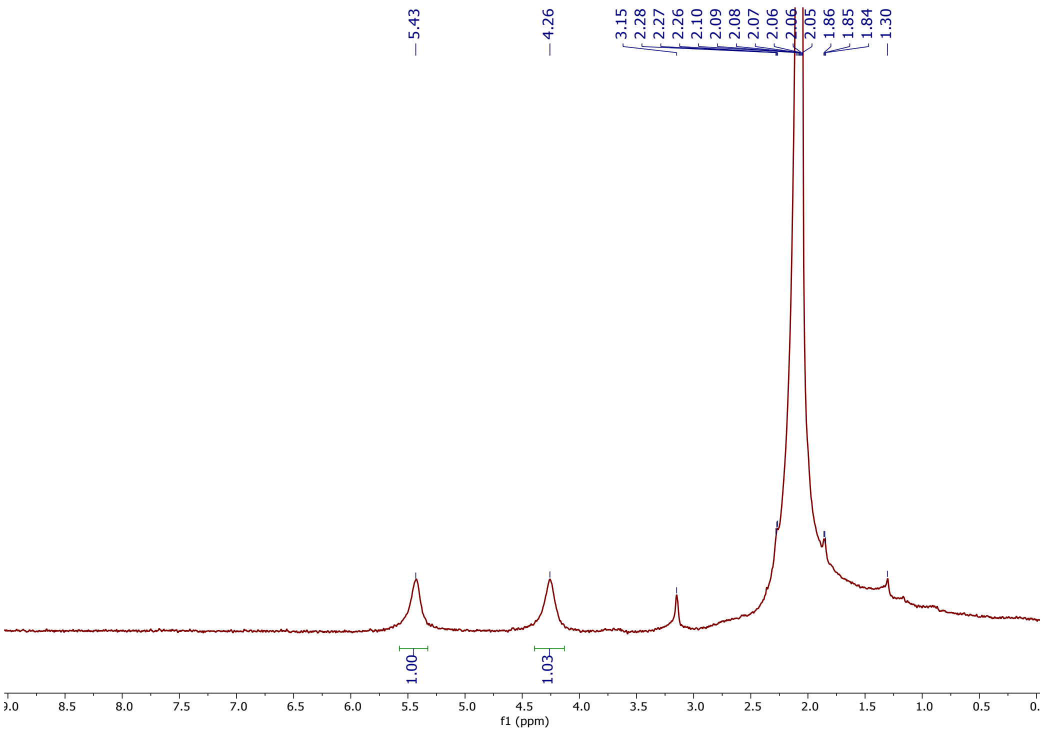

Figure S13. ^{1}H-NMR of Cs[**Cl_{10}-1**].

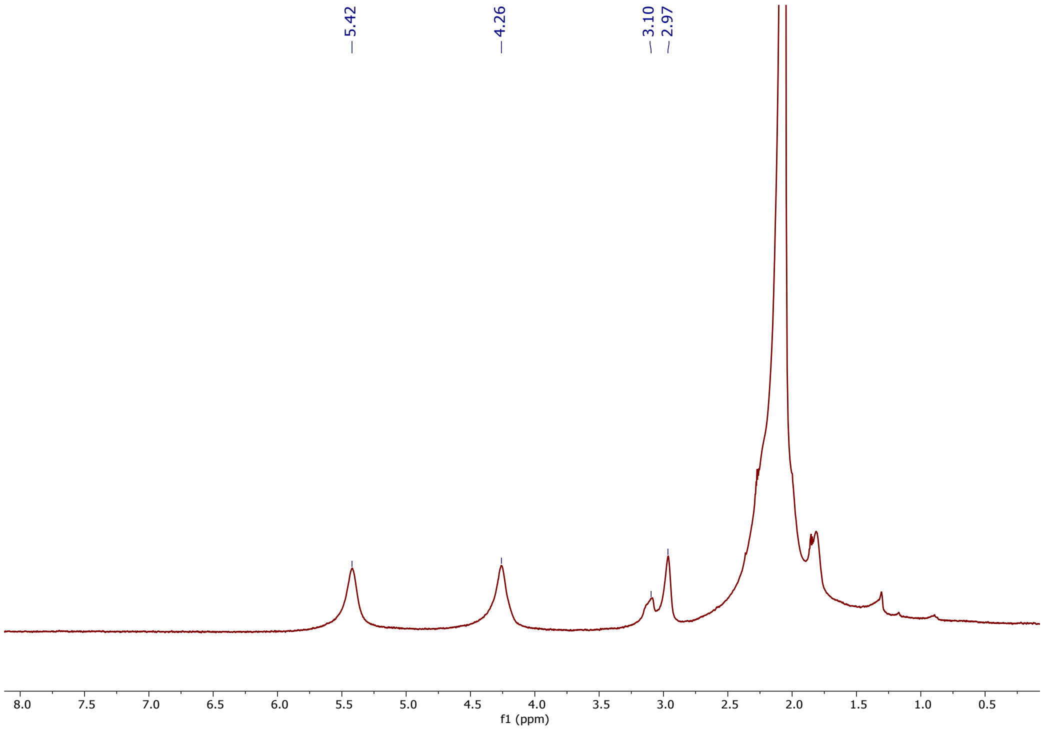

Figure S14. ^{1}H{^{11}B}-NMR of Cs[**Cl_{10}-1**].

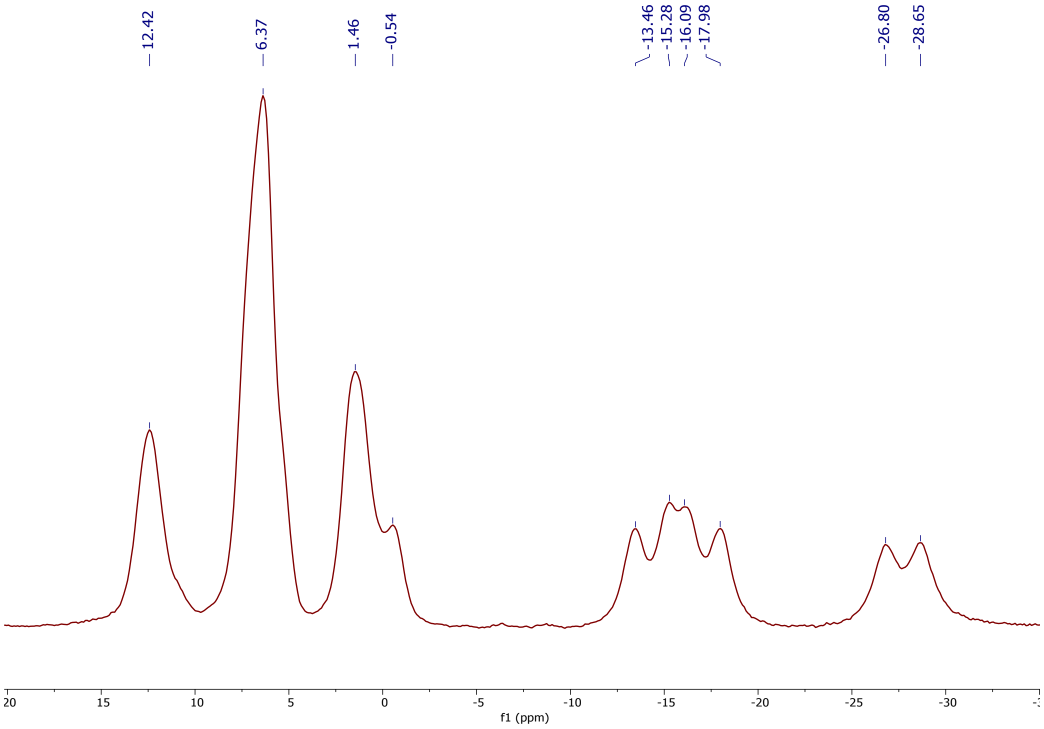

Figure S15. ^{11}B-NMR of Cs[**Cl$_{10}$-1**].

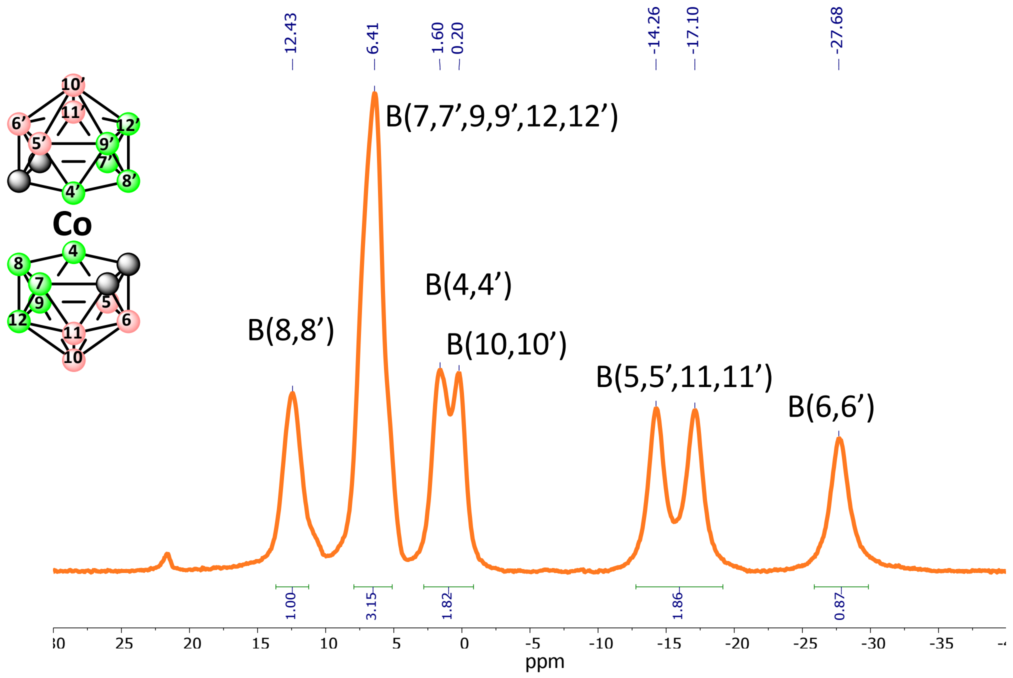

Figure S16. ^{11}B{^{1}H}-NMR of Cs[**Cl$_{10}$-1**].

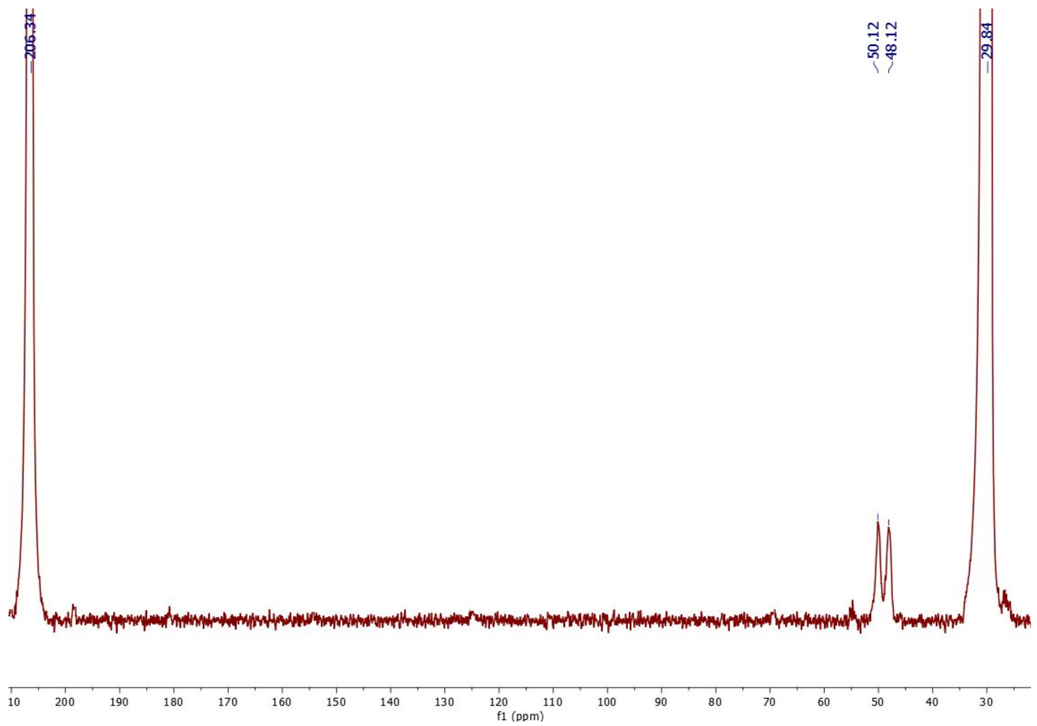

Figure S17. $^{13}C\{^{1}H\}$-NMR of Cs[$\mathbf{C_{10}}$-**1**].

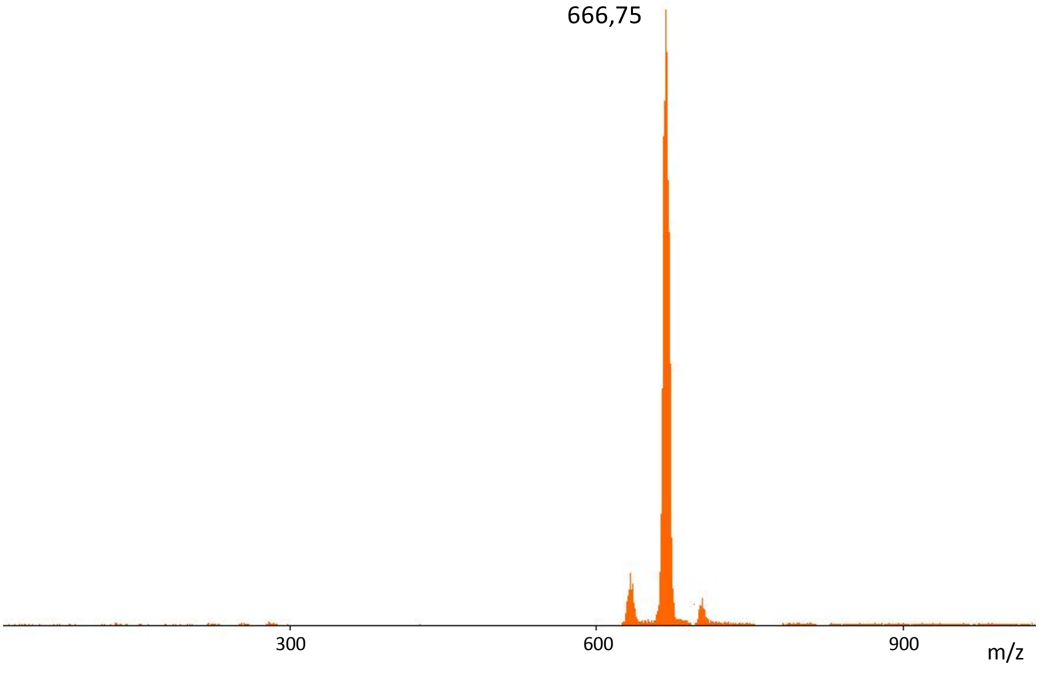

Figure S18. MALDI-TOF-MS of Cs[$\mathbf{Cl_{10}}$-**1**].

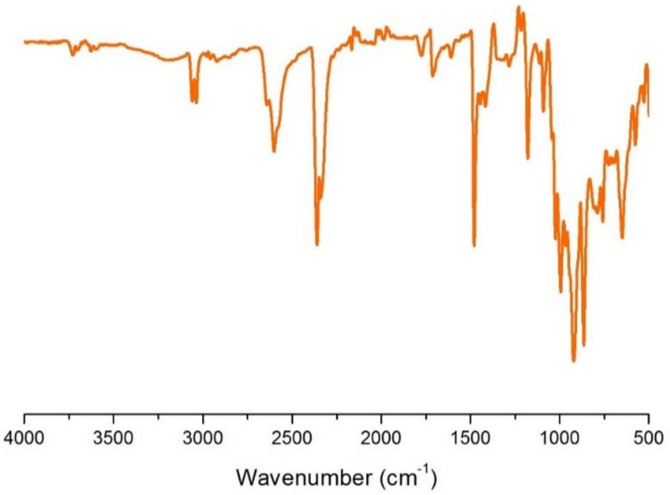

Figure S19. IR spectrum of Cs[$\mathbf{Cl_{10}}$**-1**].

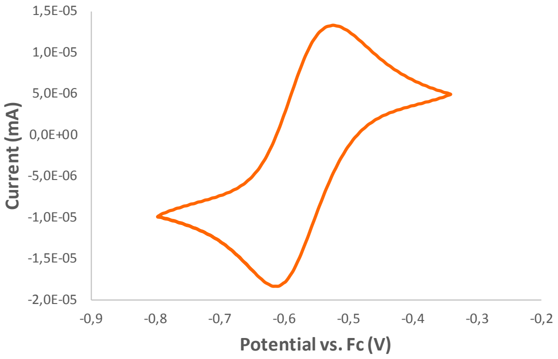

Figure S20. Cyclic voltammetry of [NMe_4][$\mathbf{Cl_{10}}$**-1**]

ORTEP representation of the crystalline structure of compound [NMe_4][$\mathbf{Cl_{10}}$**-1**]

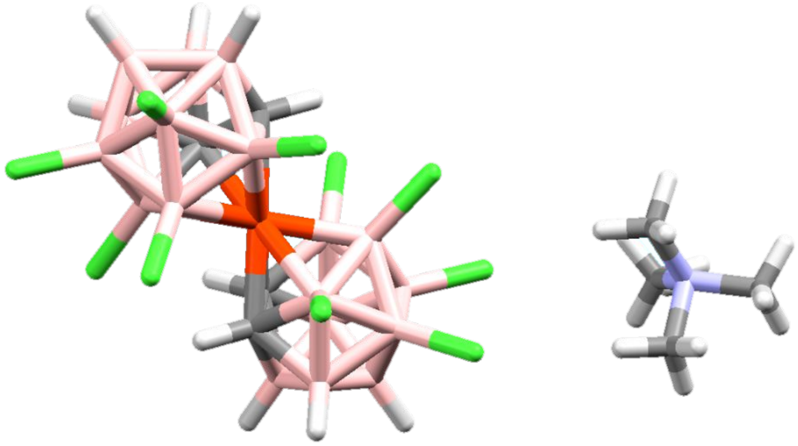

Figure S21. Representation of the crystalline structure [NMe_4][$\mathbf{Cl_{10}}$**-1**].

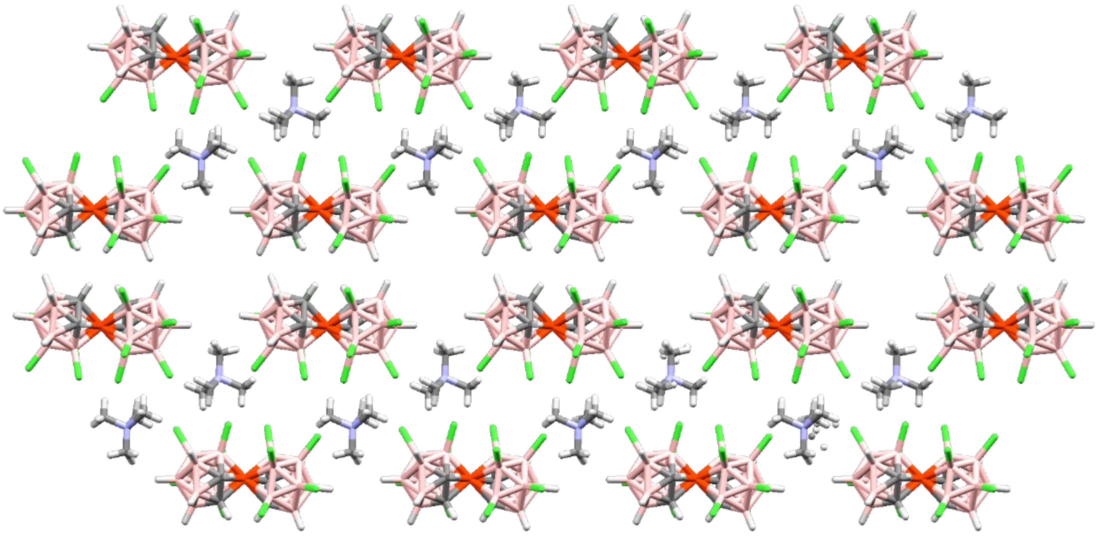

Figure S22. Crystal packaging across the a-axis

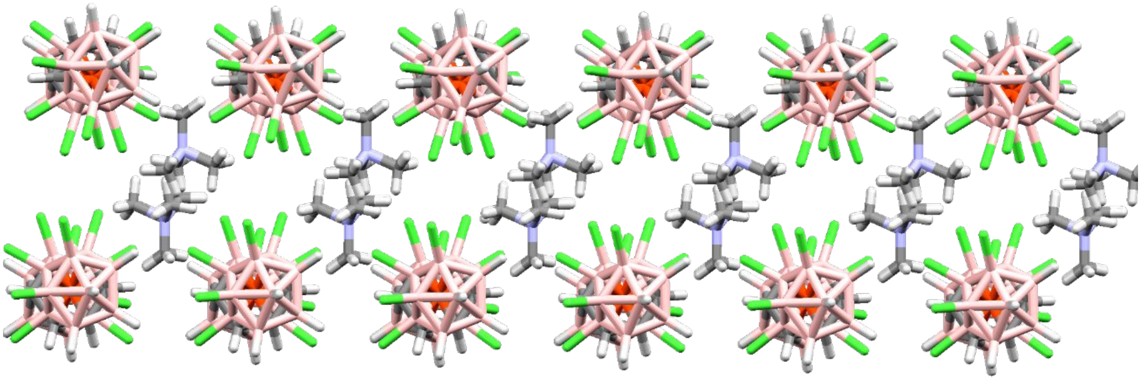

Figure S23. Crystal packaging across the b-axis.

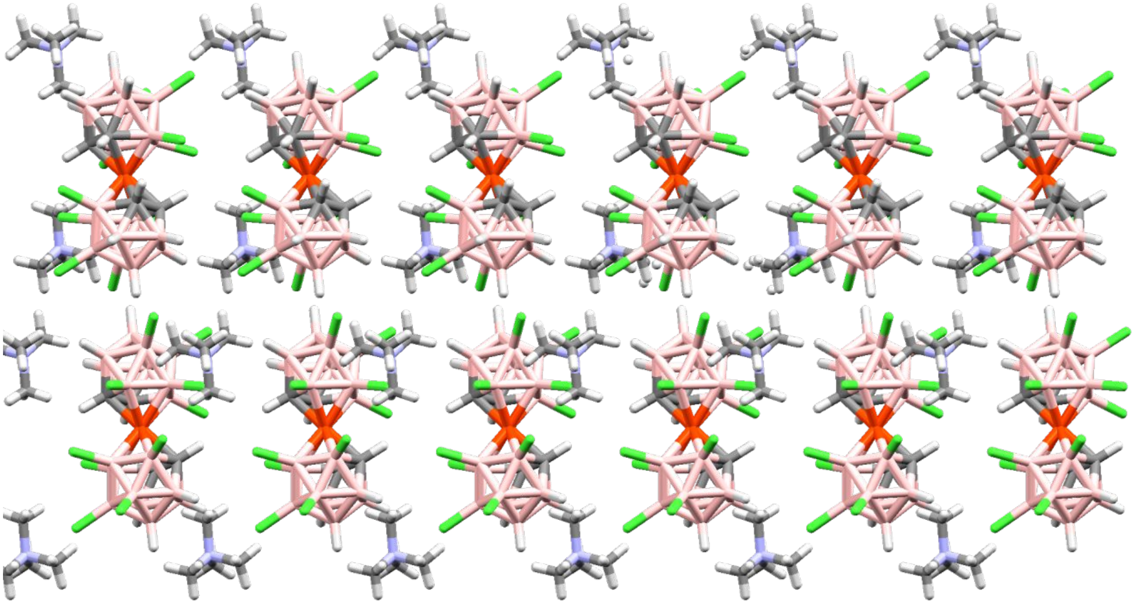

Figure S24. Crystal packaging across the c-axis.

2.4. Characterization of Cs[3,3'-Co(4,7,8,9,10,12-Cl_6-1,2-$C_2B_9H_5$)$_2$] (Cs[Cl_{12}-1]).

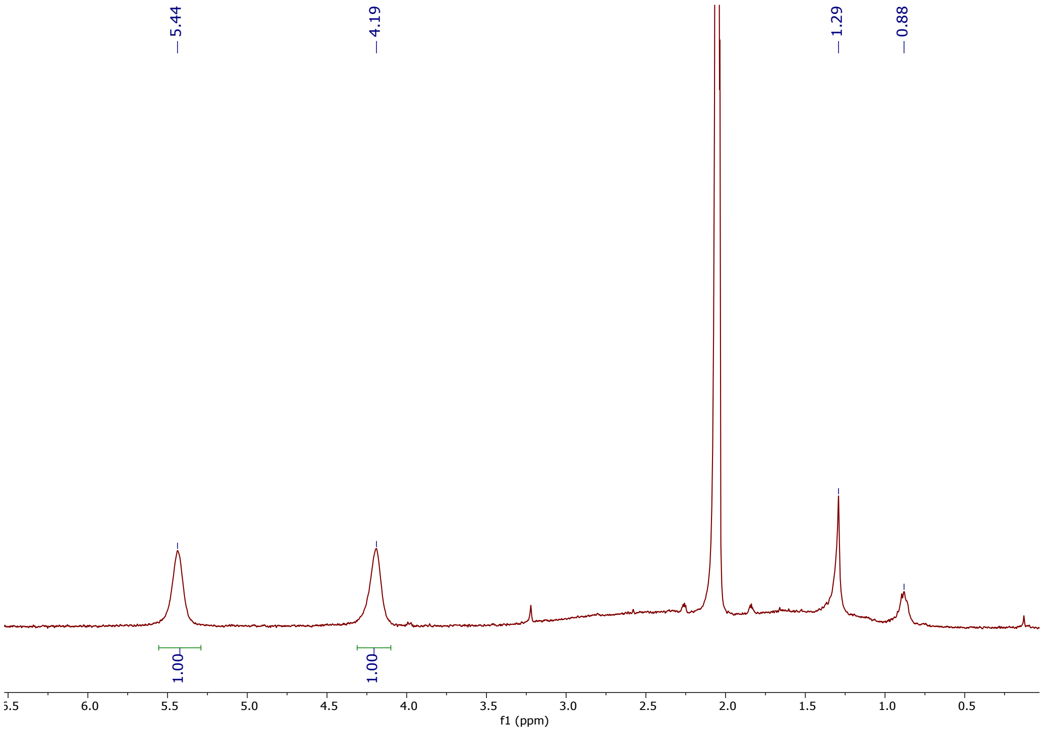

Figure S25. ^{1}H-NMR of Cs[**Cl_{12}-1**].

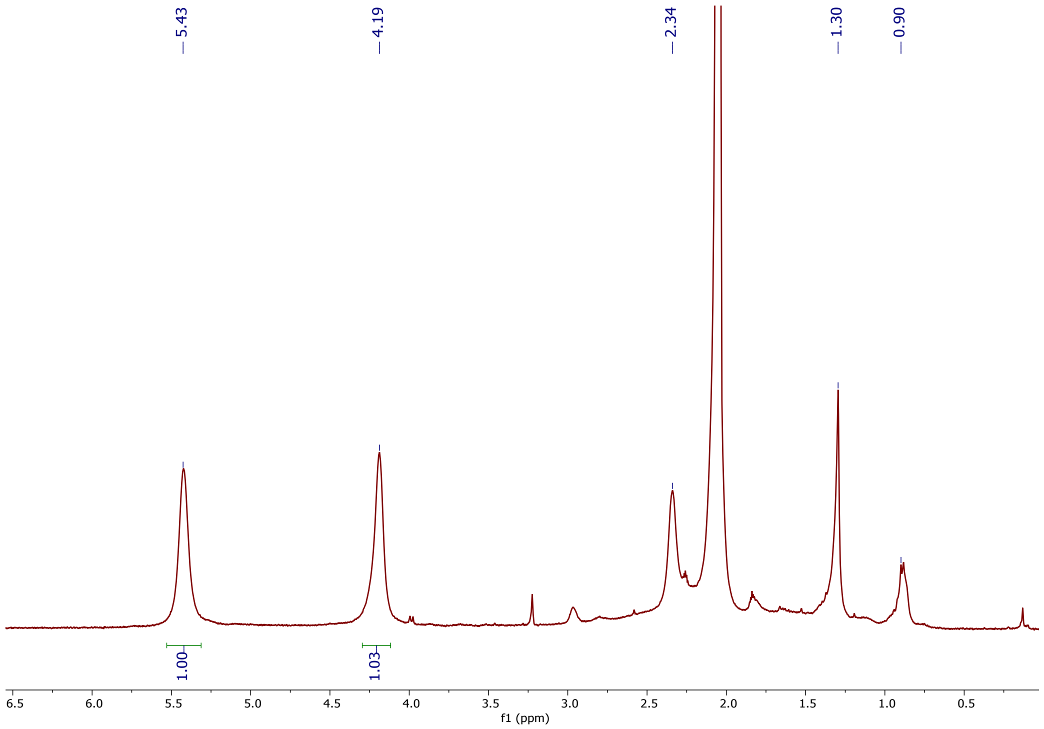

Figure S26. ^{1}H{^{11}B}-NMR of Cs[**Cl_{12}-1**].

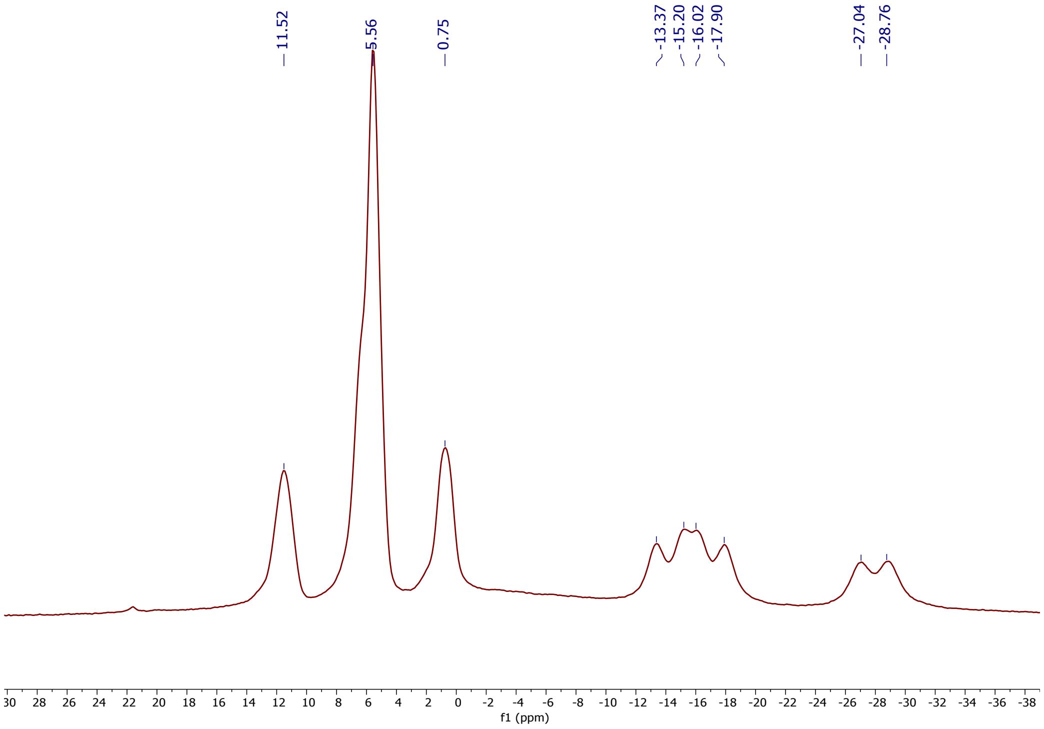

Figure S27. ^{11}B-NMR of Cs[**Cl$_{12}$-1**].

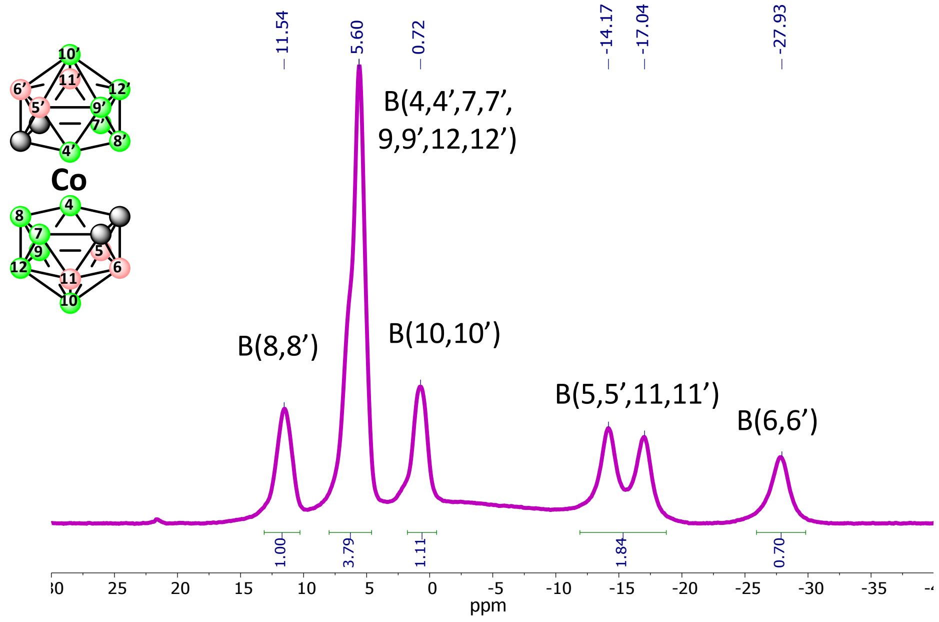

Figure S28. ^{11}B{^{1}H}-NMR of Cs[**Cl$_{12}$-1**].

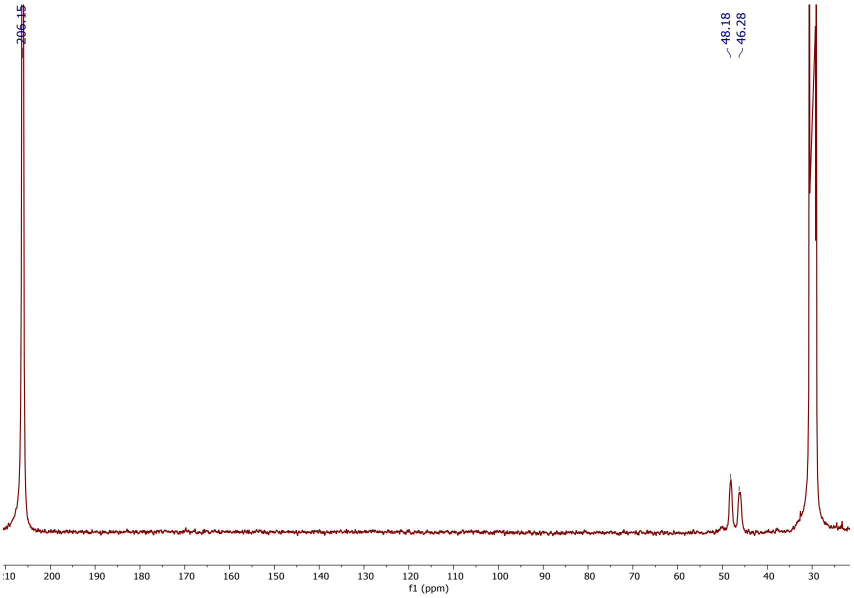

Figure S29. $^{13}C\{^{1}H\}$-NMR of Cs[**Cl_{12}-1**].

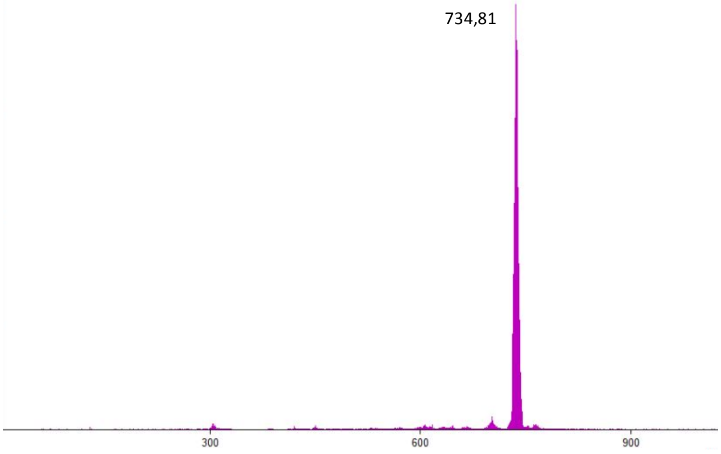

Figure S30. MALDI-TOF-MS of Cs[**Cl_{12}-1**].

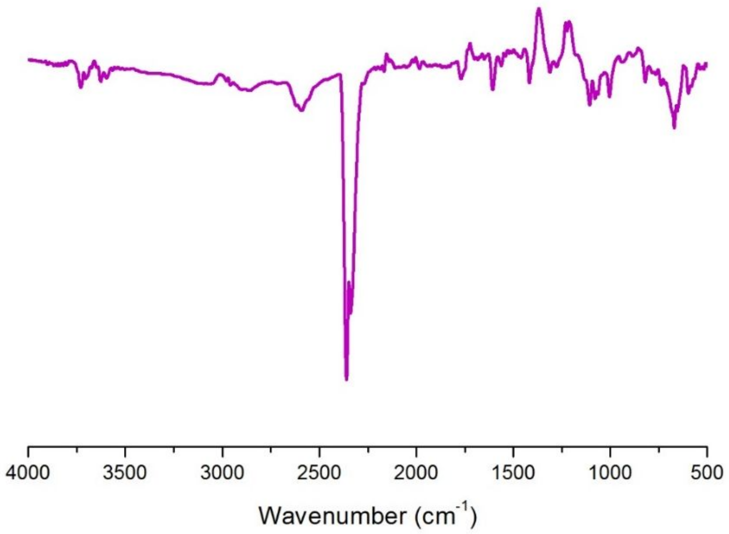

Figure S31. IR spectrum of Cs[**Cl_{12}-1**].

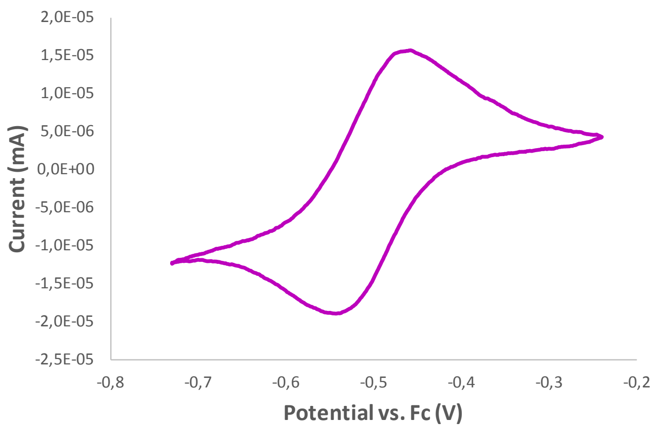

Figure S32. Cyclic voltammetry of Cs[**Cl_{12}-1**]

ORTEP representation of the crystalline structure of compound Cs[**Cl_{12}-1**] [$(CsCo(C_2B_9H_5Cl_6)_2)_2CH_2Cl_2$]. The crystal show an orange needle-like of dimensions of 0.020 x 0.040 x 0.200 mm. The crystal present a P1 21/n 1 space group with a monoclinic crystallization system. Dimension (Å) of the unit cell are: **a** 16057, **b** 7.911, **c** 24.35; with angles (º): **α** 90, **β** 98.39, **γ** 90. The total volume of the unit cell is 3158 $Å^3$. The R value of the crystal is 8.96%

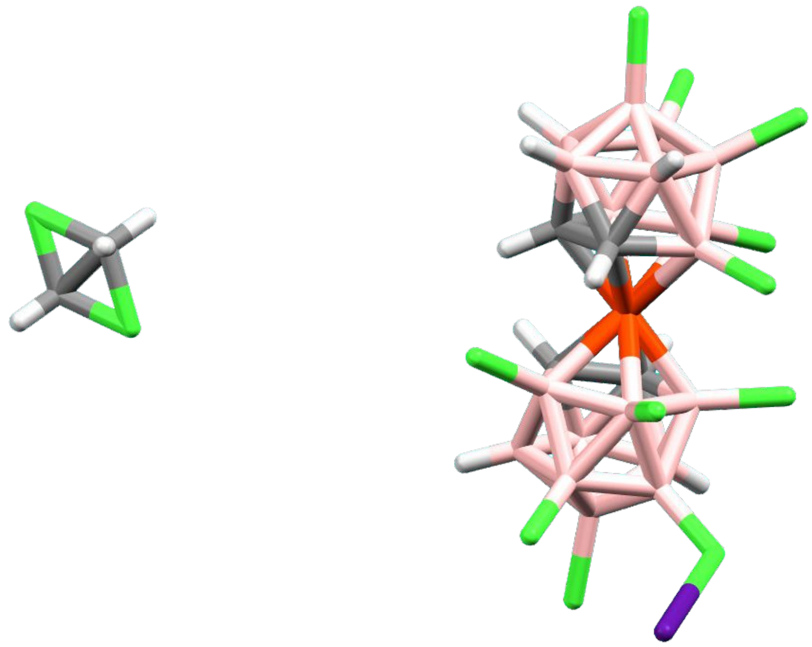

Figure S33. Crystalline structure of Cs[**Cl_{12}-1**].

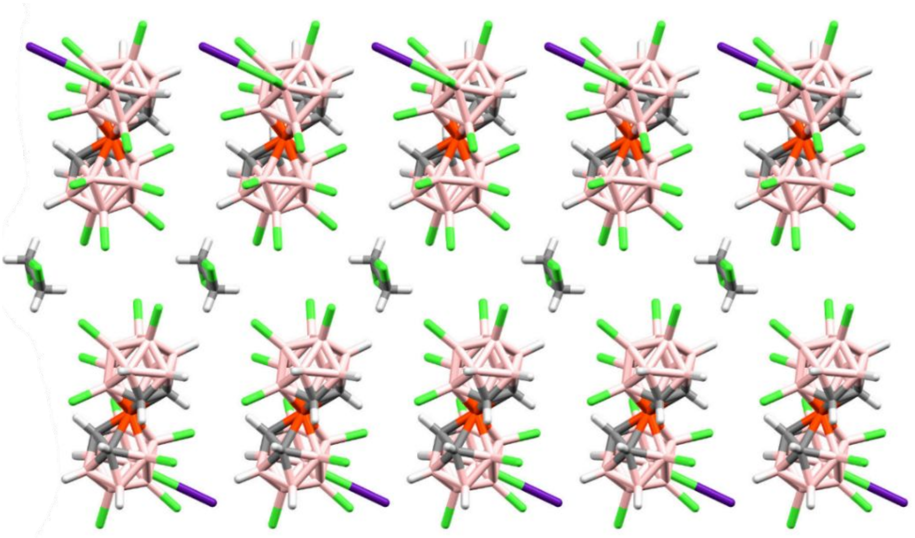

Figure S34. Crystal packaging across the a-axis

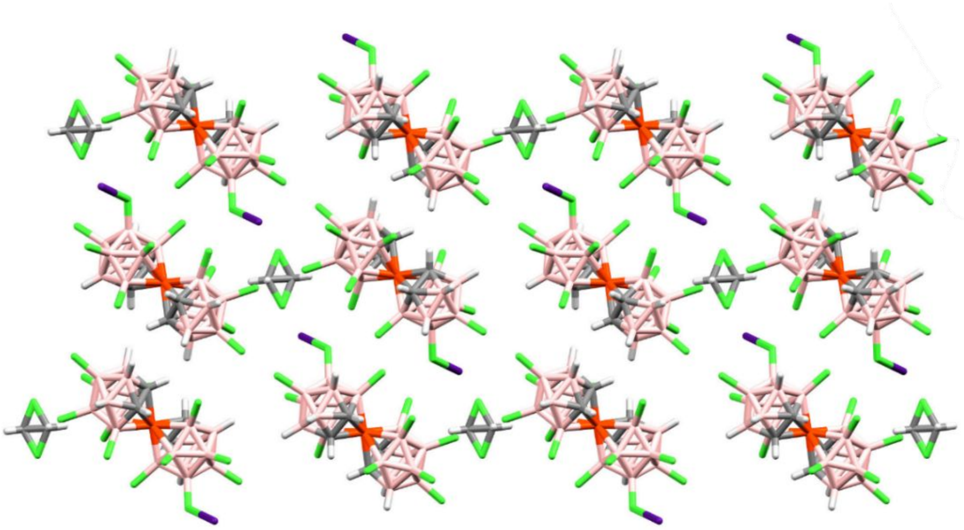

Figure S35. Crystal packaging across the b-axis.

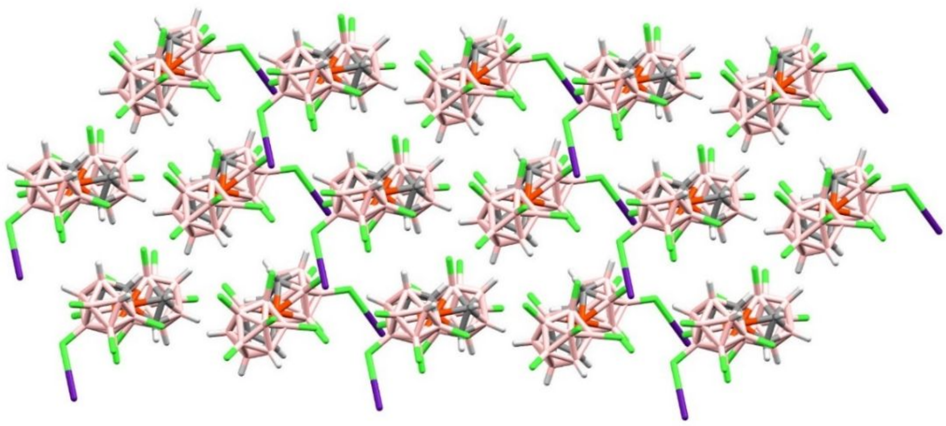

Figure 36. Crystal packaging across the c-axis.

2.5.Synthesis and characterization of $[NMe_4][3,3'\text{-}Co\text{-}(4,7\text{-}Cl_2\text{-}1,2\text{-}C_2B_9H_9)_2]$

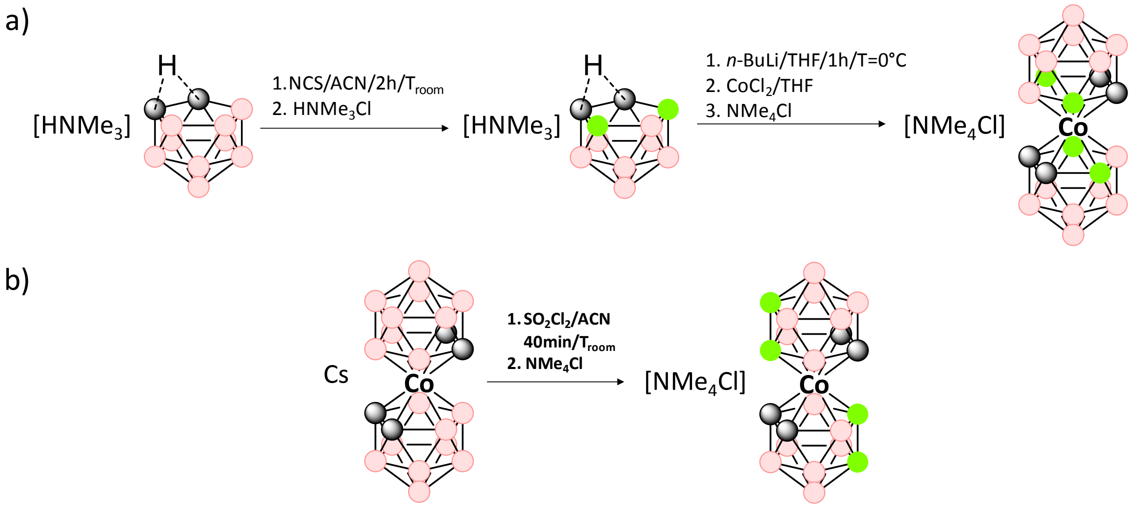

Scheme S1. a) Reaction conditions for the synthesis of $[NMe_4][3,3'\text{-}Co(4,7\text{-}Cl_2\text{-}1,2\text{-}C_2B_9H_9)_2]$ based on the literature.[1] b) Reaction conditions for the synthesis of $[NMe_4][3,3'\text{-}Co(8,9\text{-}Cl_2\text{-}1,2\text{-}C_2B_9H_9)_2]$ found in the literature.[5]

200mg of $[NMe_4]$[*nido*-9,11-Cl_2-7,8-$C_2B_9H_{10}$] (0.73 mmol) was dissolved in 5 ml of dry THF under nitrogen atmosphere and cold at 5ºC with an ice-water bath for a half-hour. 1.12 ml of *n*-BuLi (2.2 mmol, 2 M) was added dropwise. Parallel that, a solution of dry $CoCl_2$ in dry THF was prepared in another flask. Then, the transparent solution of ligand was transferred to the suspension of $CoCl_2$ and the reaction was stirred under reflux at inert atmosphere overnight. Then, the system was open and led to reflux during 2h in order to oxidise the initially Co(II) complex to Co(III) compound in presence of air. Once the reaction is finished, the solvent was removed in vacuum, and the product was extracted with 5 ml of diethyl ether and 5 ml of HCl (0.1 M) three times. The organic phase was evaporated; the brown solid was dissolved in 3 ml of water and precipitated using an aqueous solution of 100 mg (0.91 mmol) of CsCl. The brown precipitate was filtrated and washing with water three times obtaining 120 mg (62 %) of $[NMe_4]$[3,3'-Co-(4,7-Cl_2-1,2-*closo*-$C_2B_9H_9$)$_2$]. MALDI-TOF: Teor. 462.13 m/z. Found 461.36 m/z.

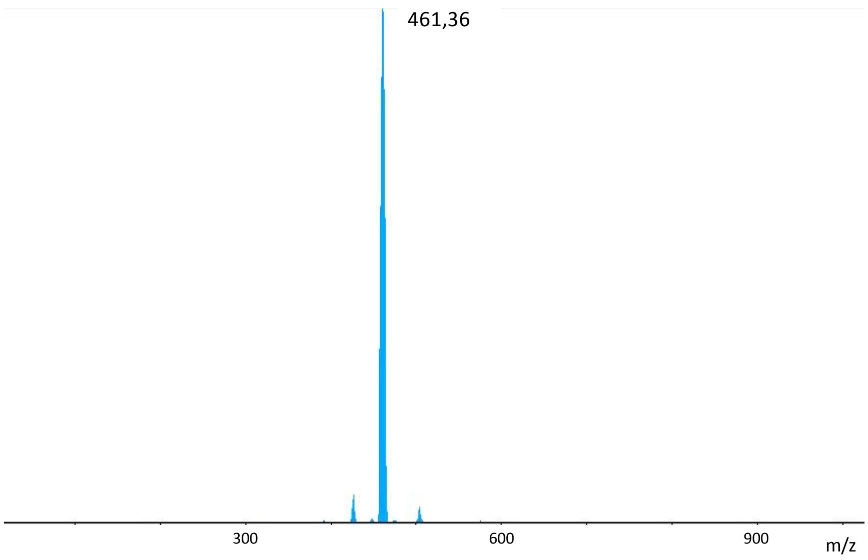

Figure S37. MALDI-TOF-MS of[NMe_4][3,3'-Co-(4,7-Cl_2-1,2-$C_2B_9H_9$)$_2$].

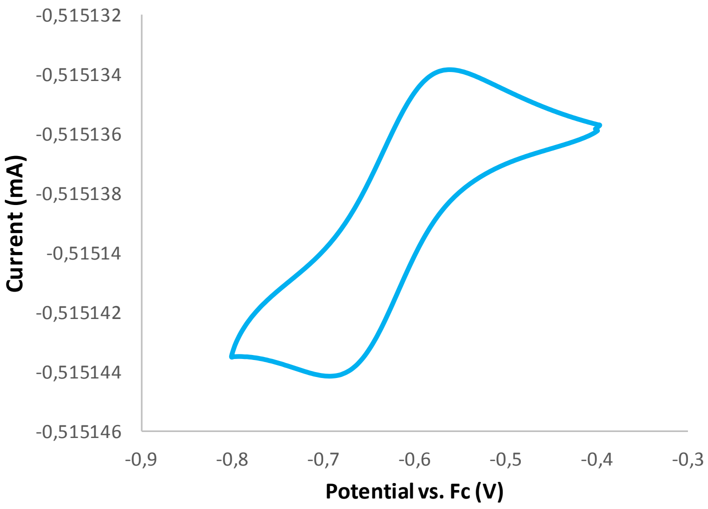

Figure S38. Cyclic voltammetry of [NMe_4][3,3'-Co-(4,7-Cl_2-1,2-$C_2B_9H_9$)$_2$].

3. Crystallographic data

	[NMe_4][**Cl_8-1**]	[NMe_4][**Cl_{10}-1**]	Cs[**Cl_{12}-1**]
**CCDC**	2087210	2087208	2087209
**Chemical formula**	$C_8H_{26}B_{18}Cl_8CoN$	$C_8H_{24}B_{18}Cl_{10}CoN$	$C_9H_{22}B_{36}Cl_{26}Co_2Cs_2$
**Formula weight**	673.41 g/mol	742.29 g/mol	1824.80 g/mol
**Temperature**	280(2) K	293(2) K	274(2) K
**Wavelength**	0.71076 Å	0.71076 Å	0.71076 Å
**Crystal size**	0.020 x 0.020 x 0.270 mm	0.080 x 0.080 x 0.370 mm	0.020 x 0.040 x 0.200 mm
**Crystal habit**	orange prism	red-orange needle	orange needle

Crystal system	orthorhombic	triclinic	monoclinic
Space group	P 21 21 21	P -1	P 1 21/n 1
Unit cell dimensions	a = 12.791(7) Å α = 90°<br>b = 14.768(8) Å β = 90°<br>c = 15.060(8) Å γ = 90°	a = 7.627(12) Å α = 104.93(7)°<br>b = 13.17(3) Å β = 95.44(5)°<br>c = 16.30(2) Å γ = 94.64(8)°	a = 16.57(2) Å α = 90°<br>b = 7.911(10) Å β = 98.39(6)<br>c = 24.35(3) Å γ = 90°
Volume	2845.(3) Å^3	1565.(5) Å^3	3158.(7) Å^3
Z	4	2	2
Density (calculated)	1.572 g/cm^3	1.574 g/cm^3	1.919 g/cm^3
Absorption coefficient	1.361 mm^{-1}	1.409 mm^{-1}	2.784 mm^{-1}
F(000)	1336	732	1724

4. References

1 E. C. Santos, A. B. Pinkerton, S. A. Kinkead, P. K. Hurlburt, S. A. Jasper, C. W. Sellers, J. C. Huffman and L. J. Todd, *Polyhedron*, 2000, **19**, 1777-1781.

2 J. Plesek, K. Base, F. Mares, F. Hanousek, B. Stibr and S. Hermanek, *Collect. Czech. Chem. Commun.*, 1984, **49**, 2776-2789.

3 Sheldrick, G. M. *Acta Cryst.* **2015**, *C71*, 3-8.

4 Sheldrick, G. M. *SADABS - Bruker Nonius scaling and absorption correction -,* Bruker AXS, Inc.: Madison, Wisconsin, USA, 2012.

5 R. Ruiz-Rosas, I. Fuentes, C. Viñas, F. Teixidor, E. Morallón and D. Cazorla-Amorós, *Sustainable Energy & Fuels*, 2018, **2**, 345-352.
